# Effective mechanical properties of multilayer nano-heterostructures

**DOI:** 10.1038/s41598-017-15664-3

**Published:** 2017-11-17

**Authors:** T. Mukhopadhyay, A. Mahata, S. Adhikari, M. Asle Zaeem

**Affiliations:** 1 Department of Engineering Science, University of Oxford, Oxford, UK; 2 Department of Materials Science and Engineering, Missouri University of Science and Technology, Rolla, USA; 3 College of Engineering, Swansea University, Swansea, UK

## Abstract

Two-dimensional and quasi-two-dimensional materials are important nanostructures because of their exciting electronic, optical, thermal, chemical and mechanical properties. However, a single-layer nanomaterial may not possess a particular property adequately, or multiple desired properties simultaneously. Recently a new trend has emerged to develop nano-heterostructures by assembling multiple monolayers of different nanostructures to achieve various tunable desired properties simultaneously. For example, transition metal dichalcogenides such as MoS_2_ show promising electronic and piezoelectric properties, but their low mechanical strength is a constraint for practical applications. This barrier can be mitigated by considering graphene-MoS_2_ heterostructure, as graphene possesses strong mechanical properties. We have developed efficient closed-form expressions for the equivalent elastic properties of such multi-layer hexagonal nano-hetrostructures. Based on these physics-based analytical formulae, mechanical properties are investigated for different heterostructures such as graphene-MoS_2_, graphene-hBN, graphene-stanene and stanene-MoS_2_. The proposed formulae will enable efficient characterization of mechanical properties in developing a wide range of application-specific nano-heterostructures.

## Introduction

**Figure 1:**
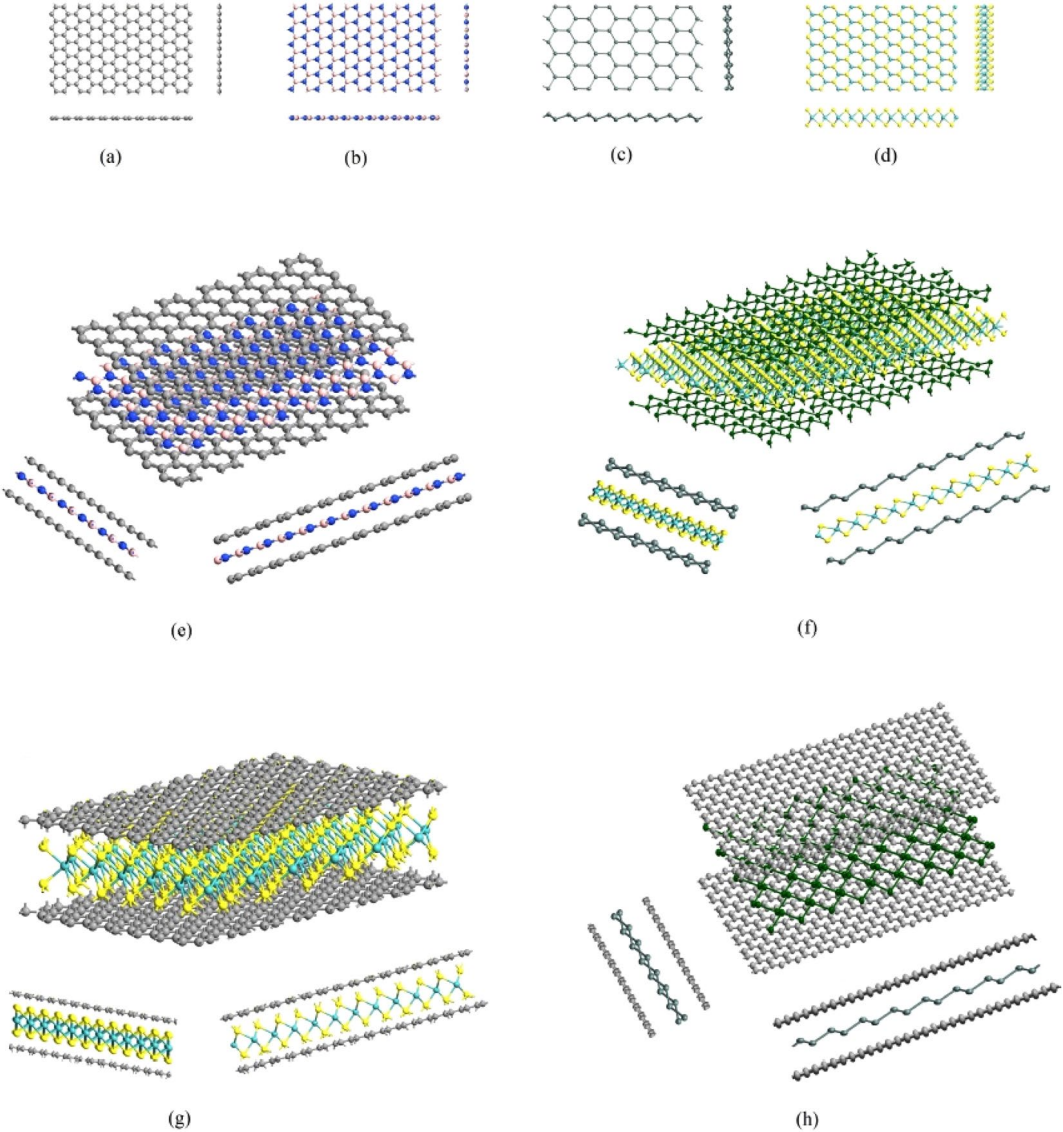
(**a**) Top view and side views of single-layer hexagonal nanostructures where all the constituent atoms are same and they are in a single plane (e.g. graphene). (**b**) Top view and side views of single-layer hexagonal nanostructures where the constituent atoms are not same but they are in a single plane (e.g. hBN, BCN). (**c**) Top view and side views of single-layer hexagonal nanostructures where the constituent atoms are same but they are in two different planes (e.g. silicene, germanene, phosphorene, stanene, borophene). (**d**) Top view and side views of single-layer hexagonal nanostructures where the constituent atoms are not same and they are in two different planes (e.g. MoS_2_, WS_2_, MoSe_2_, WSe_2_, MoTe_2_). (**e**) Three dimensional view and side views of heterostructures consisted of only monoplanar layers of materials (such as graphene-hBN heterostructures). (**f**) Three dimensional view and side views of heterostructures consisted of only multiplanar layers of materials (such as stanene-MoS_2_ heterostructures). (**g**,**h**) Three dimensional view and side views of heterostructures consisted of both monoplanar and multiplanar layers of materials (such as graphene-MoS_2_ and graphene-stanene heterostructures).

A generalized analytical approach is presented to derive closed-form formulae for the effective in-plane elastic moduli of hexagonal multiplanar nano-structures and heterostructures. Hexagonal nano-structural forms are common in various two-dimensional and quasi-two-dimensional materials. The fascinating properties of graphene^1^, a two-dimensional allotrope of carbon with hexagonal nanostructure, has led to an enormous interest and enthusiasm among the concerned scientific community for investigating more prospective two-dimensional and quasi-two-dimensional materials that could possess interesting electronic, optical, thermal, chemical and mechanical characteristics^2–4^. The interest in such hexagonal two-dimensional materials has expanded over the last decade from hBN, BCN, graphene oxides to Chalcogenides like MoS_2_, MoSe_2_ and other forms of two-dimensional materials like stanene, silicene, sermanene, phosphorene, borophene etc.^5,6^. Among these two-dimensional materials, hexagonal honeycomb-like nano-structure is a prevalent structural form^3^. Four different classes of single-layer materials with hexagonal nano-structure exist from a geometrical point of view, as shown in Fig. 1(a–d). For example, graphene^7^ consists of a single type of atom (carbon) to form a honeycomb-like hexagonal lattice structure in a single plane, while there is a different class of materials that possess hexagonal monoplanar nanostructure with different constituent atoms such as hBN^8^, BCN^9^ etc. Unlike these monoplanar hexagonal nanostructures, there are plenty of other materials that have the atoms placed in multiple planes to form a hexagonal top view. Such multiplanar hexagonal nanostructures may be consisted of either a single type of atom (such as stanene^10,11^, silicene^11,12^, germanene^11,12^, phosphorene^13^, borophene^14^ etc.), or different atoms (such as MoS_2_^15^, WS_2_^16^, MoSe_2_^17^, WSe_2_^16^, MoTe_2_^18^ etc.). Even though these two-dimensional materials show promising electronic, optical, thermal, chemical and mechanical characteristics for exciting future applications, a single nanomaterial may not possess a particular property adequately, or multiple desired properties simultaneously. To mitigate this lacuna, recently a new trend has emerged to develop nano-heterostructures by assembling multiple monolayers of different nanostructures for achieving various tunable desired properties simultaneously.

Although the single-layer of two-dimensional materials have hexagonal lattice nano-structure (top-view) in common, their out-of-plane lattice characteristics are quite different, as discussed in the preceding paragraph. Subsequently, these materials exhibit significantly different mechanical and electronic properties. For example, transition metal dichalcogenides such as MoS_2_ show exciting electronic and piezoelectric properties, but their low in-plane mechanical strength is a constraint for any practical application. In contrast, graphene possesses strong in-plane mechanical properties. Moreover, graphene is extremely soft in the out-of-plane direction with a very low bending modulus, whereas the bending modulus of MoS_2_ is comparatively much higher, depending on their respective single-layer thickness^19^. Having noticed that graphene and MoS_2_ possess such complementary physical properties, it is a quite rational attempt to combine these two materials in the form of a graphene-MoS_2_ heterostructure, which could exhibit the desired level of electronic properties and in-plane as well as out-of-plane strengths. Besides intense research on different two dimensional hexagonal nano-structural forms, recently the development of novel application-specific heterostructures has started receiving considerable attention from the scientific community due to the tremendous prospect of combining different single layer materials in intelligent and intuitive ways to achieve several such desired physical and chemical properties^20–26^.

The hexagonal nano-heterostructures can be broadly classified into three categories based on structural configuration, as shown in Fig. 1: heterostructure containing only mono-planar nanostructures (such as graphene-hBN heterostructure)^22,23,27^, heterostructure containing both mono-planar and multi-planar nanostructures (such as graphene-MoS_2_ heterostructure^19,21^, graphene-stanene heterostructure^24^, phosphorene-graphene heterostructure^28^, phosphorene-hBN heterostructure^28^, multi-layer graphene-hBN-TMDC heterostructure^26^) and heterostructure containing only multi-planar nanostructures (such as stanene-MoS_2_ heterostructure^25^, MoS_2_-WS_2_ heterostructure^20^). Recently different forms of multi-layer heterostructures have started receiving immense attention from the scientific community for showing interesting chemical, thermal, optical, electronic and transport properties^24,25,29,30^. Even though the heterostructures show various exciting physical and chemical characteristics, effective mechanical properties such as Young’s moduli and Poisson’s ratios are of utmost importance for accessing the viability in application of such nano-heterostructures in various nanoelectromechanical systems. The research in this field is still in a very nascent stage and investigations on elastic properties of these built-up structural forms are very scarce to find in literature^20,21^.

The common practises to investigate these nanostructures are first principle studies/ab-initio and molecular dynamics, which can reproduce the results of experimental analysis with the cost of computationally expensive and time consuming supercomputing facilities. Moreover, availability of interatomic potentials can be a practical barrier in carrying out molecular dynamics simulation for nano-heterostructures, which are consisted of multiple materials. The accuracy of molecular dynamics simulation depends on the interatomic potentials and the situation can become worse in case of nano-heterostructures due to the possibility of having lesser accuracy for built-up structural forms. Molecular mechanics based analytical closed form formulae are presented by many researchers for materials having hexagonal nano-structures in a single layer such as graphene, hBN, stanene, MoS_2_ etc.^7,8,31–33^. This approach of mechanical property characterization for single-layer nanostructures is computationally very efficient, yet accurate and physically insightful. However, the analytical models concerning two-dimesional hexagonal nano-structures developed so far are limited to single-layer structural forms; development of efficient analytical approaches has not been attempted yet for nano-heterostructures. Considering the future prospect of research in this field, it is essential to develop computationally efficient closed-form formulae for the elastic moduli of nano-hetrostructures that can serve as a ready reference for the researchers without the need of conducting expensive and time consuming molecular dynamics simulations or laboratory experiments. This will accelerate the process of novel material development based on the application-specific need of achieving multiple tunable properties simultaneously to a desirable extent.

In this article, we aim to address the strong rationale for developing a generalized compact analytical model leading to closed-form and high fidelity expressions for characterizing the mechanical properties of a wide range of hexagonal nano-heterostructures. Elastic properties of four different heterostructures (graphene-hBN, graphene-MoS_2_, graphene-stanene and stanene-MoS_2_), belonging to all the three classes as discussed in the preceding paragraphs, are investigated considering various stacking sequences. The analytical formulae for elastic moduli of heterostructures are applicable to any number of different constituent single-layer materials with multi-planar or mono-planar hexagonal nanostructures.

## Results

### Closed-form analytical formulae for the elastic moduli of heterostructures

In this section, the closed-form analytical expressions of elastic moduli for generalized multiplaner hexagonal nano-heterostructures are presented. The molecular mechanics based approach for obtaining the equivalent elastic properties of atomic bonds is well-documented in scientific literature^31,34,35^. Besides that the mechanics of mono-planar hexagonal honeycomb-like structure is found to be widely investigated across different length scales^36–40^. Therefore, the main contribution of this article lies in proposing computationally efficient and generalized analytical formulae for nano-heterostructures (having constituent single-layer materials with monoplanar and multiplanar structural form) and thereby presenting new results for various stacking sequence of different nano-heterostructures belonging to the three different classes as described in the preceding section (graphene-MoS_2_, graphene-hBN, graphene-stanene and stanene-MoS_2_).

**Figure 2:**
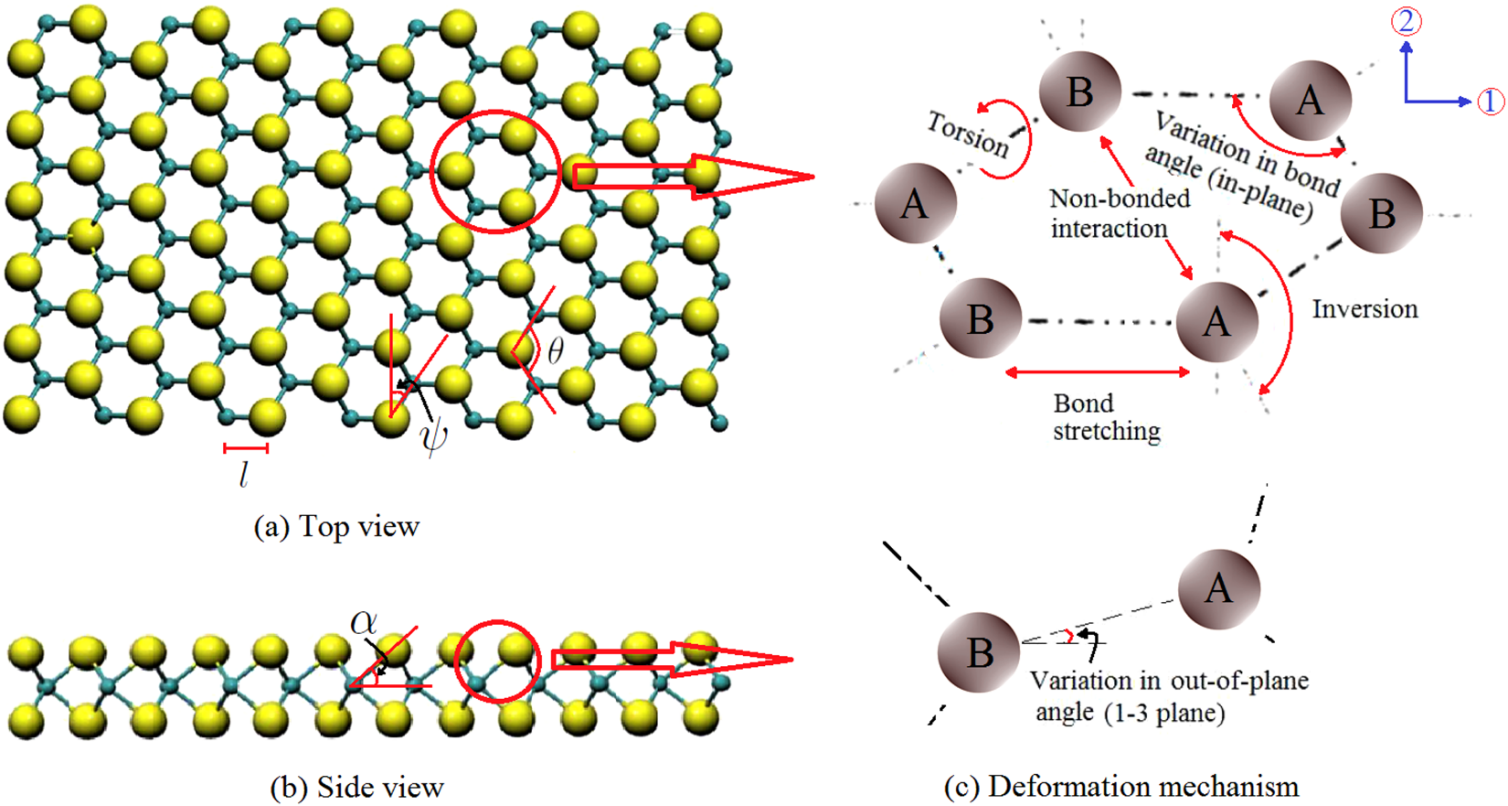
(**a**,**b**) Top view and side view of a generalized form of multiplanar hexagonal nano-structure. (The in-plane angles *θ* and *ψ* are indicated in Fig. 2(a), wherein it is evident that $\psi ={90}^{\circ }-\frac{\theta }{2}$. The out-of-plane angle *α* is indicated in Fig. 2(b)). (**c**) Energy components associated with the in-plane (1–2 plane) and out-of-plane (1–3 plane) deformation mechanisms (Direction 1 and 2 are indicated in the figure. Direction 3 is perpendicular to the 1–2 plane. Here A and B indicate two different atoms).

For atomic level behaviour of nano-scale materials, the total interatomic potential energy can be expressed as the sum of various individual energy terms related to bonding and non-bonding interactions^34^. Total strain energy (*E*) is expressed as the sum of energy contributions from bending of bonds (*E*_*b*_), bond stretching (*E*_*s*_), torsion of bonds (*E*_*t*_) and energies associated with non-bonded terms (*E*_*nb*_) such as the van der Waals attraction, the core repulsions and the coulombic energy (refer to Fig. 2).

1 $$E={E}_{s}+{E}_{b}+{E}_{t}+{E}_{nb}$$

However, among all the energy components, effect of bending and stretching are predominant in case of small deformation^31,35^. For the multiplanar hexagonal nano-structures (such as stanene and MoS_2_), the strain energy caused by bending consists of two components, in-plane component (*E*_*bI*_) and out-of-plane component (*E*_*bO*_). The out-of-plane component becomes zero for monoplanar nanostructures such as graphane and hBN. Thus the total interatomic potential energy (*E*) can be expressed as

2 $$\begin{array}{ll}E & =\,{E}_{s}+{E}_{bI}+{E}_{bO}\\  & =\,\frac{1}{2}{k}_{r}{({\rm{\Delta }}l)}^{2}+\frac{1}{2}{k}_{\theta }{({\rm{\Delta }}\theta )}^{2}+\frac{1}{2}{k}_{\theta }{({\rm{\Delta }}\alpha )}^{2}\end{array}$$

where Δ*l*, Δ*θ* and Δ*α* denote the change in bond length, in-plane and out-of-plane angle respectively. The quantities *k*_*r*_ and *k*_*θ*_ represents the force constants for bond stretching and bending respectively. The molecular mechanics parameters (*k*_*r*_ and *k*_*θ*_) and structural mechanics parameters (*EA* and *EI*) of a uniform circular beam with cross-sectional area *A*, length *l*, Young’s modulus *E*, and second moment of area *I*, are related as: ${K}_{r}=\frac{EA}{l}$ and ${k}_{\theta }=\frac{EI}{l}$ ^31,34,35^. Based on this relationship, the closed form expressions for the effective elastic moduli of multilayer hexagonal nano-heterostructures are derived following a multi-stage idealization scheme using force equilibrium and deformation compatibility conditions. The closed form expressions for the two in-plane Young’s moduli of nano-heterostructures are derived as

3 $${E}_{1}=\frac{1}{t}\sum _{i=1}^{n}\frac{\cos \,{\psi }_{i}}{\mathrm{(1}+\,\sin \,{\psi }_{i})(\frac{{l}_{i}^{2}}{12{k}_{\theta i}}({\sin }^{2}{\psi }_{i}+{\cos }^{2}{\psi }_{i}{\sin }^{2}{\alpha }_{i})+\frac{{\cos }^{2}{\psi }_{i}\,{\cos }^{2}{\alpha }_{i}}{{k}_{ri}})}$$

4 $${E}_{2}=\frac{1}{t}\sum _{i=1}^{n}\frac{1+\,\sin \,{\psi }_{i}}{\cos \,{\psi }_{i}(\frac{{l}_{i}^{2}}{12{k}_{\theta i}}({\cos }^{2}{\psi }_{i}+{\sin }^{2}{\psi }_{i}{\sin }^{2}{\alpha }_{i}+2\,{\sin }^{2}{\alpha }_{i})+\frac{{\cos }^{2}{\alpha }_{i}}{{k}_{ri}}({\sin }^{2}{\psi }_{i}+2))}$$

The subscript *i* in the above expressions indicates the molecular mechanics and geometrical properties (as depicted in Fig. 2(a,b)) corresponding to *i* ^*th*^ layer of the heterostructure. The overall thickness of the heterostructure is denoted by *t*. *n* represents the total number of layers in the heterostructure. Expressions for the two in-plane Poisson’s ratios are derived as

5 $${\nu }_{12}=\frac{{\sum }_{i=1}^{n}\frac{\cos \,{\psi }_{i}}{\mathrm{(1}+\,\sin \,{\psi }_{i})(\frac{{l}_{i}^{2}}{12{k}_{\theta i}}({\sin }^{2}{\psi }_{i}+{\cos }^{2}{\psi }_{i}\,{\sin }^{2}{\alpha }_{i})+\frac{{\cos }^{2}{\psi }_{i}\,{\cos }^{2}{\alpha }_{i}}{{k}_{ri}})}}{{\sum }_{i=1}^{n}\frac{12{k}_{\theta i}}{\sin \,{\psi }_{i}\,\cos \,{\psi }_{i}\,{\cos }^{2}{\alpha }_{i}{l}_{i}^{2}}}$$

6 $${\nu }_{21}=\frac{{\sum }_{i=1}^{n}\frac{1+\,\sin \,{\psi }_{i}}{\cos \,{\psi }_{i}(\frac{{l}_{i}^{2}}{12{k}_{\theta i}}({\cos }^{2}{\psi }_{i}+{\sin }^{2}{\psi }_{i}\,{\sin }^{2}{\alpha }_{i}+2\,{\sin }^{2}{\alpha }_{i})+\frac{{\cos }^{2}{\alpha }_{i}}{{k}_{ri}}({\sin }^{2}{\psi }_{i}+2))}}{{\sum }_{i=1}^{n}\frac{12{k}_{\theta i}}{\sin \,{\psi }_{i}\,\cos \,{\psi }_{i}\,{\cos }^{2}{\alpha }_{i}{l}_{i}^{2}}}$$

Here *ν*_12_ and *ν*_21_ represent the in-plane Poisson’s ratios for loading directions 1 and 2 respectively. Thus the elastic moduli of a hexagonal nano-heterostructure can be obtained using the closed-form analytical formulae (Equations 3–6) from molecular mechanics parameters (*k*_*r*_ and *k*_*θ*_), bond length (*l*), in-plane bond angle (*ψ*) and out-of-plane angle (*α*), which are well-documented in the molecular mechanics literature. The analytical formulae are valid for small deformation of the structure (i.e. the linear region of stress-strain curve). The effect of inter-layer stiffness contribution due Lennard-Jones potentials are found to be negligible for the in-plane elastic moduli considered in this study and therefore, neglected in the analytical derivation (refer to section 7 of the supplementary material).

### Validation and analytical predictions for the elastic moduli of heterostructures

Results are presented for the effective elastic moduli of hexagonal multi-layer nano-heterostructures based on the formulae proposed in the preceding section. As investigations on nano-heterostructures is a new and emerging field of research, the results available for the elastic moduli of different forms of heterostructures is very scarce in scientific literature. We have considered four different nano-heterostructures to present the results: graphene-MoS_2_, graphene-hBN, graphene-stanene and stanene-MoS_2_ (belonging to the three categories as depicted in the introduction section). Though all these four heterostructures have received attention from the concerned scientific community for different physical and chemical properties recently, only the graphene-MoS_2_ heterostructure has been investigated using molecular dynamics simulation for the Young’s modulus among all other elastic moduli^20,21^. Thus we have validated the proposed analytical formulae for Young’s moduli of graphene-MoS_2_ heterostructure with available results from literature. New results are presented for the two in-plane Poisson’s ratios of graphene-MoS_2_ heterostructure using the analytical formulae, which are validated by carrying out separate molecular dynamics simulations. Having the developed analytical formulae validated for the two Young’s moduli and Poisson’s ratios, new results are provided for the other three considered heterostructures accounting for the effect of stacking sequence. Moreover, it can be noted that for single layer of the heterostructure (i.e. for *n* = 1), the proposed analytical formulae can be used to predict the effective elastic moduli of monoplanar (i.e. *α* = 0) and multiplanar (i.e. *α* ≠ 0) materials. The analytical predictions for the Young’s moduli and Poisson’s ratios of such single-layer materials are further validated with reference results from literature, as available.

As shown in Tables 1–5, in the case of single-layer hexagonal nanostructures (*n* = 1) belonging to all the four classes as described in the preceding section (graphene, hBN, stanene and MoS_2_), the in-plane Young’s moduli obtained using the proposed analytical formulae are in good agreement with reported values in literature for graphene, hBN, stanene and MoS_2_. These observations corroborate the validity of the proposed analytical formulae in case of a single-layer. However, in case of Poisson’s ratios, the reported values in scientific literature for graphene and hBN show wide range of variability, while the reference values of Poisson’s ratios for stanene and MoS_2_ are very scarce in available literature. The results predicted by the proposed formulae agree well with most of the reported values for Poisson’s ratios.

**Table 1:** Results for two Young’s moduli (*E*_1_ and *E*_2_, in TPa) and two in-plane Poisson’s ratios (*ν*_12_ and *ν*_21_) of graphene-MoS_2_ (G–M) heterostructure with different stacking sequences (The results obtained using the proposed formulae are compared with the existing results from literature, as available.

Configuration	Present results		Reference (*E*_1_ = *E*_2_)	Present results		Reference (*ν*_12_ = *ν*_21_)
	*E*_1_	*E*_2_		*ν*_12_	*ν*_21_	
G	1.0419	1.0419	1.05^19^, 1 ± 0.1^67^	0.2942	0.2942	0.34^68^, 0.195^69^
G/G	1.0419	1.0419	1.06^19^, 1.04 ± 0.1^70^	0.2942	0.2942	0.2798 [MD]
M	0.1778	0.3549	0.16^19^, 0.27 ± 0.1^71^	0.0690	0.1376	0.1019 [MD], 0.21^72^
M/M	0.1778	0.3549	0.27^19^, 0.2 ± 0.1^71^	0.0690	0.1376	0.1018 [MD]
G/M	0.4893	0.6025	0.53^19^, 0.49 ± 0.05^20^	0.1672	0.2059	0.2153 [MD]
G/M/G	0.6357	0.7189	0.68^19^, 0.56^21^	0.2058	0.2328	0.1805 [MD]
M/G/M	0.3678	0.5059	0.45^19^	0.1318	0.1813	0.1859 [MD]

However, as the Poisson’s ratios for the heterostructures are not available in literature, we have conducted molecular dynamics (MD) simulations for the Poisson’s ratios. The thickness of single layer of graphene and MoS_2_ are considered as 0.34 nm and 0.6033 nm, respectively).

**Table 2:** Results for two in-plane Young’s moduli (*E*_1_ and *E*_2_, in TPa) and two in-plane Poisson’s ratios (*ν*_12_ and *ν*_21_) of graphene-hBN (G–H) heterostructure with different stacking sequences (The thickness of single layer of graphene and hBN are considered as 0.34 nm and 0.33 nm, respectively).

Configuration	*E*_1_	*E*_2_	*ν*_12_	*ν*_21_
G	1.049	1.049	0.2942	0.2942
G/G	1.049	1.049	0.2942	0.2942
H	0.8056	0.8056	0.2901	0.2901
H/H	0.8056	0.8056	0.2901	0.2901
G/H	0.9255	0.9255	0.2925	0.2925
G/H/G	0.9647	0.9647	0.2931	0.2931
H/G/H	0.8859	0.8859	0.2918	0.2918

**Table 3:** Results for two in-plane Young’s moduli (*E*_1_ and *E*_2_, in TPa) and two in-plane Poisson’s ratios (*ν*_12_ and *ν*_21_) of graphene-stanene (G–S) heterostructure with different stacking sequences (The thickness of single layer of graphene and stanene are considered as 0.34 nm and 0.172 nm, respectively).

Configuration	*E*_1_	*E*_2_	*ν*_12_	*ν*_21_
G	1.049	1.049	0.2942	0.2942
G/G	1.049	1.049	0.2942	0.2942
S	0.3166	0.3736	0.1394	0.1645
S/S	0.3166	0.3736	0.1394	0.1645
G/S	0.7982	0.8174	0.2563	0.2625
G/S/G	0.8955	0.9070	0.2726	0.2761
S/G/S	0.6771	0.7058	0.2333	0.2432

**Table 4:** Results for two in-plane Young’s moduli (*E*_1_ and *E*_2_, in TPa) and two in-plane Poisson’s ratios (*ν*_12_ and *ν*_21_) of stanene-MoS_2_ (S–M) heterostructure with different stacking sequences (The thickness of single layer of stanene and MoS_2_ are considered as 0.172 nm and 0.6033 nm, respectively).

Configuration	*E*_1_	*E*_2_	*ν*_12_	*ν*_21_
S	0.3166	0.3736	0.1394	0.1645
S/S	0.3166	0.3736	0.1394	0.1645
M	0.1778	0.3549	0.0690	0.1376
M/M	0.1778	0.3549	0.0690	0.1376
S/M	0.2086	0.3591	0.0831	0.1430
S/M/S	0.2282	0.3617	0.0925	0.1466
M/S/M	0.1951	0.3573	0.0768	0.1406

**Table 5:** Results for Young’s moduli (TPa) and Poisson’s ratios of single-layer hexagonal nanostructures).

Material	Present Results	Reference results from literature (*E*_1_ = *E*_2_ and *ν*_12_ = *ν*_21_)
Graphene	*E*_1_ = 1.0419	1.00 ± 0.1 TPa^67^, 1.05 TPa^73,74^, 1.041 TPa^31^
	*E*_2_ = 1.0419	1.00 ± 0.1 TPa^67^, 1.05 TPa^73,74^, 1.041 TPa^31^
	*ν*_12_ = 0.2942	0.34^68^, 0.17^74^, 0.41^75^, 0.195^69^, 0.653–0.848^7^
	*ν*_21_ = 0.2942	0.34^68^, 0.17^74^, 0.41^75^, 0.195^69^, 0.653–0.848^7^
hBN	*E*_1_ = 0.8056	0.76 ± 0.045^76^, 0.821^77^, 0.842^78^, 0.815^79^
	*E*_2_ = 0.8056	0.76 ± 0.045^76^, 0.821^77^, 0.842^78^, 0.815^79^
	*ν*_12_ = 0.2901	0.2–0.3^80^, 0.2–0.24^80^, 0.384–0.389^8^, 0.384–0.389^8^, 0.211^8^, 0.2–0.4^81^
	*ν*_21_ = 0.2901	0.2–0.3^80^, 0.2–0.24^80^, 0.384–0.389^8^, 0.384–0.389^8^, 0.211^8^, 0.2–0.4^81^
Stanene	*E*_1_ = 0.3166	0.307^56^
	*E*_2_ = 0.3736	0.307^56^
		—
	*ν*_21_ = 0.1645	—
MoS_2_	*E*_1_ = 0.1778	0.27 ± 0.099 TPa^71^, 0.233 TPa^82^, 0.248 TPa^83^
	*E*_2_ = 0.3549	0.27 ± 0.099 TPa^71^, 0.233 TPa^82^, 0.248 TPa^83^
		0.21^72^, 0.29^63^
	*ν*_21_ = 0.1376	0.21^72^, 0.29^63^

Table 1 presents the value of two Young’s moduli obtained from the proposed analytical formulae for nano-heterostructures considering different stacking sequences of graphene and MoS_2_. The results are compared with the numerical values reported in scientific literature. It can be noted that the difference between *E*_1_ and *E*_2_ is not recognized in most of the previous investigations and the results presented as *E*_1_ = *E*_2_. The Young’s moduli *E*_1_ and *E*_2_ are found to be different for multiplanar single-layer nanostructural forms (such as stanene and MoS_2_). A similar trend has been reported before by Li^41^ for MoS_2_. Thus the effective Young’s moduli of the heterostructures with at least one layer of multiplanar structural form is expected to exhibit different *E*_1_ and *E*_2_ values. In Table 1 it can be observed that for single and bi-layer of graphene *E*_1_ = *E*_2_, while for single and bi-layer of MoS_2_ *E*_1_ ≠ *E*_2_. In case of heterostructures consisting of both graphene and MoS_2_ the value of *E*_2_ is observed to be higher than *E*_1_. However, the numerical values of *E*_1_ for different stacking sequences are found to be in good agreement with the values of Young’s modulus reported in literature (presumably obtained for direction-1) corroborating the validity of the developed closed-form expressions. We have carried out separate molecular dynamics simulations for graphene – MoS_2_ heterostructures to validate the analytical predictions of Poisson’s ratios, as Poisson’s ratios have not been reported for graphene–MoS_2_ heterostructures in literature. The analytical predictions of Poisson’s ratios reported in Table 1 are found to be in good agreement with the results of molecular dynamics simulations. Similar to the results of Young’s moduli for graphene-MoS_2_ heterostructure, the two in-plane Poisson’s ratios (*ν*_12_ and *ν*_21_) are found to have different values when at least one multi-planar structural form is present in the heterostructure. Thus having the analytical formulae for all the elastic moduli validated, we have provided new results for three other nano-heterostructures in the following paragraphs based on Equations 3–6.

Table 2 provides the results for elastic moduli of graphene-hBN heterostructure considering different stacking sequences. It is observed that the two Young’s moduli and two in-plane Poisson’s ratios are equal (i.e. *E*_1_ = *E*_2_ and *ν*_12_ = *ν*_21_) in case of graphene-hBN heterostructure as these are consisted of only mono-planar structural forms. Table 3 presents the results for elastic moduli of graphene-stanene heterostructure considering different stacking sequences. As stanene has a multi-planar structural form, the two Young’s moduli and two in-plane Poisson’s ratios show different values (i.e. *E*_1_ ≠ *E*_2_ and *ν*_12_ ≠ *ν*_21_) when at least one of the constituent layers of the heterostructure is stanene. Table 4 presents the results for elastic moduli of stanene-MoS_2_ heterostructure considering different stacking sequences. As stanene and MoS_2_ both have multi-planar structural form, the two Young’s moduli and two in-plane Poisson’s ratios show considerably different values (i.e. *E*_1_ ≠ *E*_2_ and *ν*_12_ ≠ *ν*_21_). The results of different elastic moduli corresponding to various stacking sequences are noticed to have an intermediate value between the respective elastic modulus for single layer of the constituent materials, as expected on a logical basis.

**Figure 3:**
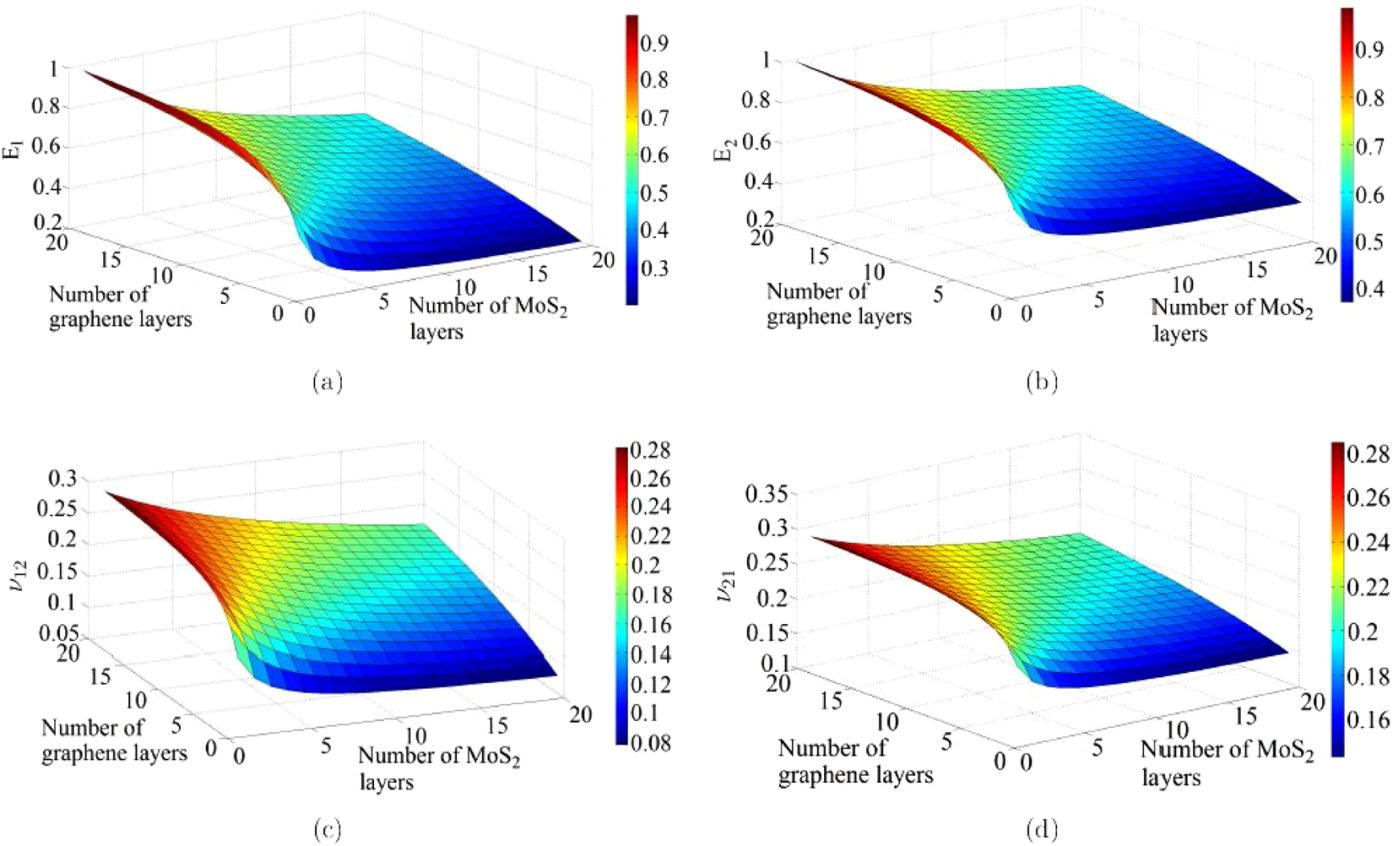
(**a**,**b**) Variation of in-plane Young’s moduli (*E*_1_ and *E*_2_) with number of layers in a graphene-MoS_2_ heterostructure. (**c**,**d**) Variation of the in-plane Poisson’s ratios (*ν*_12_ and *ν*_21_) with number of layers in a graphene-MoS_2_ heterostructure.

**Figure 4:**
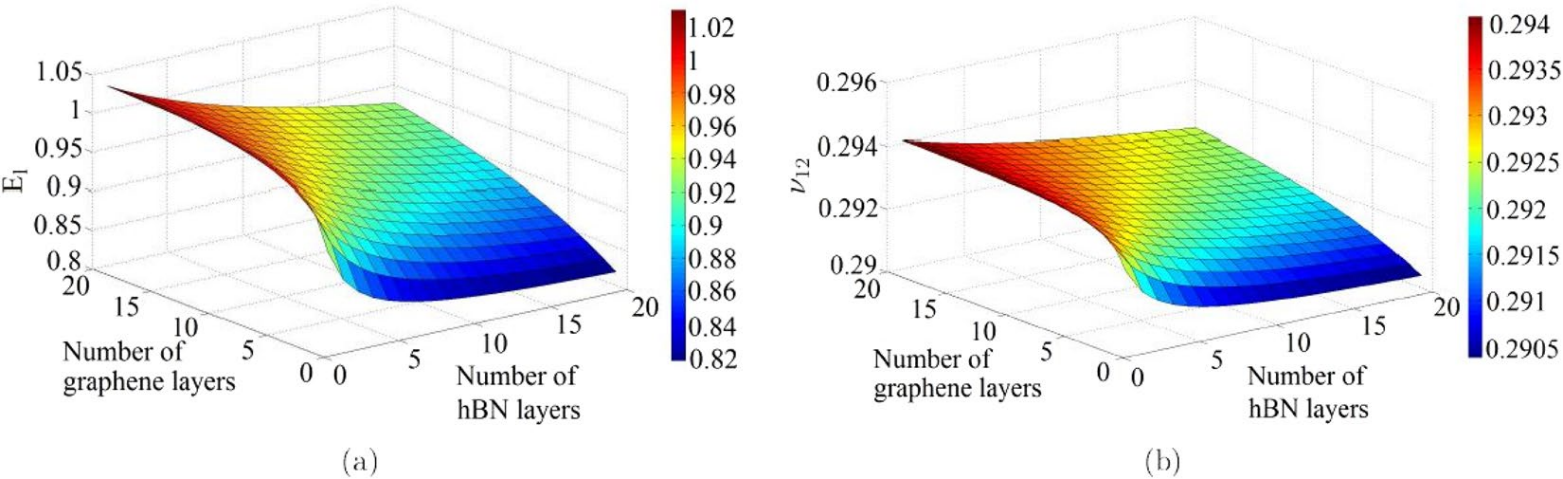
(**a**) Variation of in-plane Young’s modulus (*E*_1_) with number of layers in a graphene-hBN heterostructure (Variation of *E*_2_ with number of layers in a graphene-hBN heterostructure is same as *E*_1_). (**b**) Variation of the in-plane Poisson’s ratio (*ν*_12_) with number of layers in a graphene-hBN heterostructure (Variation of *ν*_21_ with number of layers in a graphene-hBN heterostructure is same as *ν*_12_).

**Figure 5:**
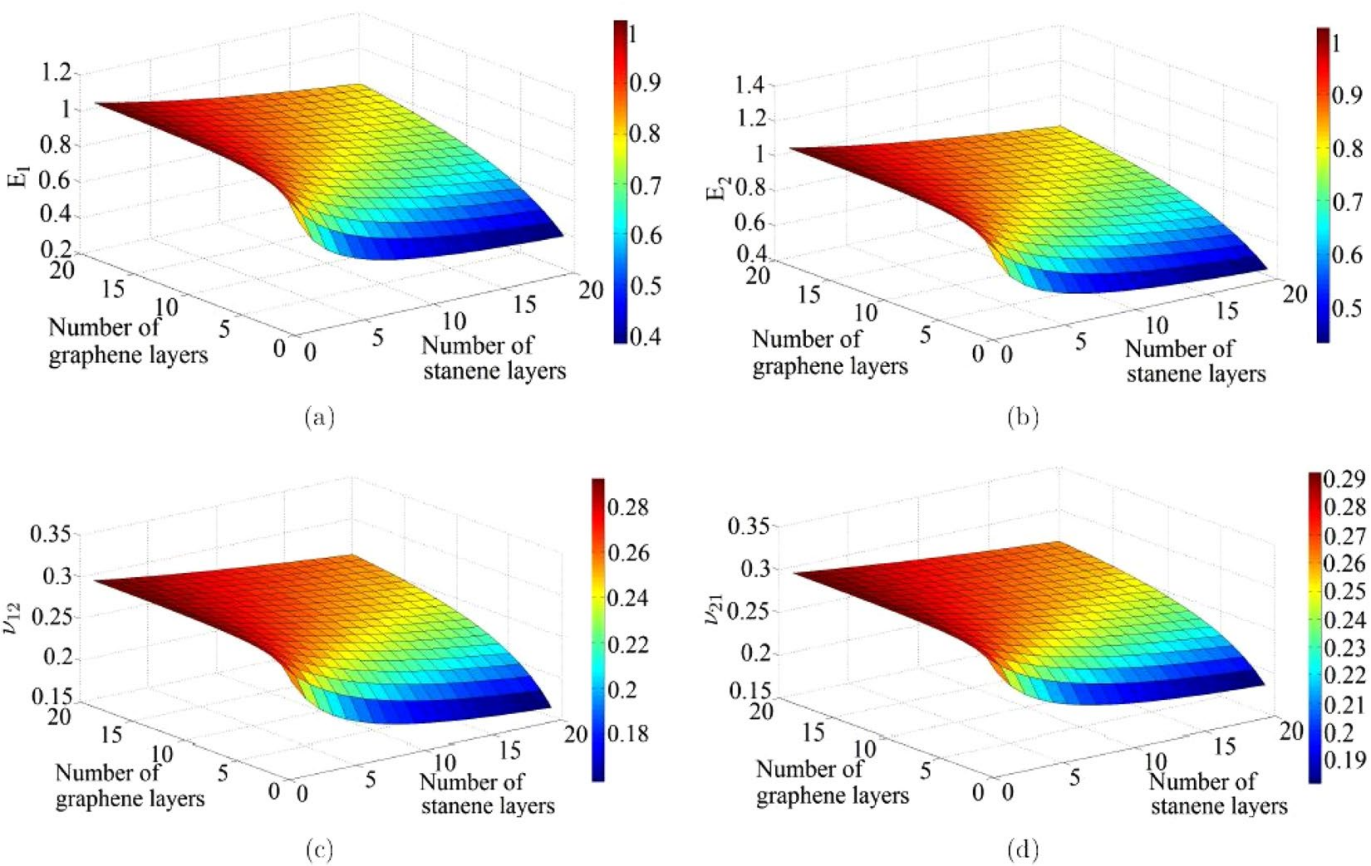
(**a**,**b**) Variation of in-plane Young’s moduli (*E*_1_ and *E*_2_) with number of layers in a graphene-stanene heterostructure. (**c**,**d**) Variation of the in-plane Poisson’s ratios (*ν*_12_ and *ν*_21_) with number of layers in a graphene-stanene heterostructure.

**Figure 6:**
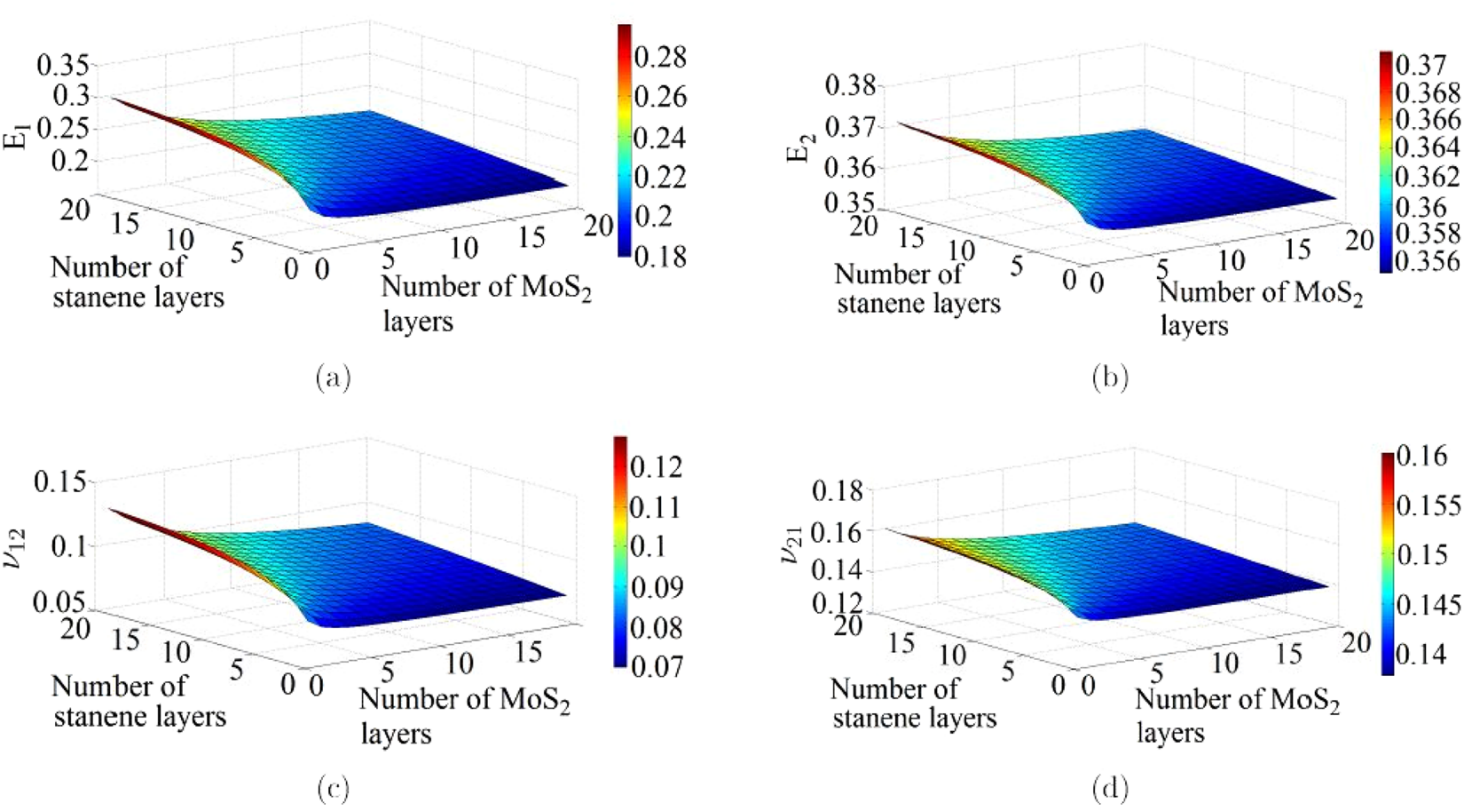
(**a**,**b**) Variation of in-plane Young’s moduli (*E*_1_ and *E*_2_) with number of layers in a stanene-MoS_2_ heterostructure. (**c**,**d**) Variation of the in-plane Poisson’s ratios (*ν*_12_ and *ν*_21_) with number of layers in a stanene-MoS_2_ heterostructure.

The physics based analytical formulae for nano-heterostructures presented in this article are capable of obtaining the elastic moduli corresponding to any stacking sequence of the constituent layer of nano-materials. However, from the expressions it can be discerned that the numerical values of elastic moduli actually depend on the number of layers of different constituent materials rather than their exact stacking sequences. From a mechanics view-point, this is because of the fact that the in-plane properties are not a function of the distance of individual constituent layers from the neutral plane of the entire heterostructure. Figures 3, 4, 5, 6 present the variation of different elastic moduli with number of layers of the constituent materials considering the four different heterostructures belonging from the three different categories, as described in the preceding section. It is observed that the trend of variation for two Young’s moduli and two in-plane Poisson’s ratios are similar for graphene-MoS_2_ and graphene-stanene heterostructures with little difference in the actual numerical values. The variation of elastic moduli for graphene-hBN heterostructure are presented for *E*_1_ and *ν*_12_ as the numerical values are exactly same for the two Young’s moduli and two in-plane Poisson’s ratios, respectively. The plots furnished in this section can readily provide an idea about the trend of variation of elastic moduli with stacking sequence of multi-layer nano-heterostructures in a comprehensive manner; exact values of the elastic moduli corresponding to various stacking sequences can be easily obtained using the proposed computationally efficient closed-form formulae.

## Discussion

We have presented computationally efficient analytical closed-form expressions for the effective elastic moduli of multi-layer nano-heterostructures, wherein individual layers may have multiplanar (i.e. *α* ≠ 0) or monoplanar (i.e. *α* = 0) configurations. It is interesting to notice that the generalized analytical formulae developed for the Young’s moduli of heterostructures can be reduced to the closed-form expressions provided by Shokrieh and Rafiee^31^ for graphene considering single-layer (i.e. *n* = 1), *α* = 0 and *ψ* = 30°.

7 $${E}_{1}={E}_{2}=\frac{4\sqrt{3}{k}_{r}{k}_{\theta }}{t(\frac{{k}_{r}{l}^{2}}{4}+9{k}_{\theta })}$$

It can be noted from the presented results that the single-layer materials having regular monoplanar hexagonal nano-structures (such as graphene and hBN) have equal value of elastic modulus in two perpendicular directions (i.e. *E*_1_ = *E*_2_ and *ν*_12_ = *ν*_21_). However, for single-layer materials with multiplanar nanostructure, the elastic modulus for direction-2 is more than that of direction-1, even though the difference is not significant. Similar observation is found to be reported in literature^41^. For single-layer of materials, the formulae of elastic moduli deduced from Equations 3–6 by replacing *n* = 1, perfectly obeys the Reciprocal theorem (i.e. *E*_1_ *ν*_21_ = *E*_2_ *ν*_12_)^42^. In case of nano-heterostructures, the Young’s moduli and Poisson’s ratios possess different values if at least any one of the layers have a material with multiplanar hexagonal nano-structure (i.e. *E*_1_ ≠ *E*_2_ and *ν*_12_ ≠ *ν*_21_). An advantage of the proposed bottom-up approach of considering layer-wise equivalent material property is that it allows us to neglect the effect of lattice mismatch in evaluating the effective elastic moduli for multi-layer heterostructures consisting of different materials. In the derivation for effective elastic moduli of such heterostructues, the deformation compatibility conditions of the adjacent layers are satisfied. This is expected to give rise to some strain energy locally at the interfaces, which is noted in previous studies^21^. From the derived expressions it can be discerned that the numerical values of elastic moduli actually depend on the number of layers of different constituent materials rather than their stacking sequences. In case of multi-layer nanostructures constituted of the layers of same material (i.e. bulk material), it can be expected from Equations 3 and 4 that the Young’s moduli would reduce due to the presence of inter-layer distances, which, in turn, increase the value of overall thickness *t*.

Effective mechanical properties such as Young’s moduli and Poisson’s ratios are of utmost importance to access the viability for the use of nano-heterostructures in various nanoelectromechanical applications. The major contribution of this work is to develop the generalized closed-form analytical formulae for multi-layer nano-heterostructures. Thses formulae are also applicable to single-layer of materials with monoplanar as well as multiplanar nanostructures. Thus the developed analytical formulae for elastic moduli can be used as an efficient reference for the entire spectrum of materials with lattice-like structural form and the heterostructures obtained by combining multiple layers of different such materials with any stacking sequence. Such generalization in the derived formulae, with the advantage of being computationally efficient and easy to implement, opens up a tremendous potential scope in the field of novel application-specific heterostructure development. We have validated the proposed expressions considering multiple stacking sequences with existing results of literature and separate molecular dynamics simulations for the Young’s moduli and Poisson’s ratios of graphene-MoS_2_ heterostructure, respectively. In-depth new results are presented for the Young’s moduli and Poisson’s ratios of three other nano-heterostructures (graphene-hBN, graphene-stanene and stanene-MoS_2_). Even though the results are presented in this article considering only two different constituent materials in a single heterostructure (such as graphene-MoS_2_, graphene-hBN, graphene-stanene and stanene-MoS_2_), the proposed formulae can be used for heterostructures containing any number of different materials^26^. The physics-based analytical formulae are capable of providing a comprehensive in-depth insight on the behaviour of such multilayer heterostructures. Noteworthy feature of the present analytical approach is the computational efficiency and cost-effectiveness compared to conducting nano-scale experiments or molecular dynamics simulations. Thus, besides deterministic analysis of elastic moduli, as presented in this paper, the efficient closed-form formulae could be an attractive option for carrying out uncertainty analysis^43–50^ based on a Monte Carlo simulation based approach (refer to section 8 of the supplementary material). The bottom-up approach based concept to develop expressions for hexagonal nano-heterostructures can be extended to other forms of nanostrcutures in future.

After several years of intensive investigation, research concerning graphene has logically reached to a rather mature stage. Thus investigation of other two dimensional and quasi-two dimensional materials have started receiving the due attention recently. However, the possibility of combining single layers of different two dimensional materials (heterostructures) has expanded this field of research dramatically; well beyond the scope of considering a simple single layer graphene or other 2D material. The interest in such heterostructures is growing very rapidly with the advancement of synthesizing such materials in laboratory^22,23^, as the interest in graphene did few years ago. The attentiveness is expected to expand further in coming years with the possibility to consider different tunable nanoelectromechanical properties of the prospective combination (single and multi-layer structures with different stacking sequences) of so many two dimensional materials. This, in turn introduces the possibility of opening a new dimension of application-specific material development that is analogous to metamaterials^51,52^ in nano-scale. The present article can contribute significantly in this exciting endeavour.

In summary, we have developed computationally efficient physics-based analytical expressions for predicting the equivalent elastic moduli of multi-layer nano-heterostructures. The proposed expressions are validated for graphene–MoS_2_ heterostructures by carrying out separate molecular dynamics simulations and available results from literature. New results are presented for graphene–hBN, graphene–stanene and stanene–MoS_2_ heterostructures using the developed analytical framework. As the proposed closed-form formulae are general in nature and applicable to wide range of materials and their combinations with hexagonal nano-structures, the present article can serve as a ready reference for characterizing the material properties in future nano-materials development.

## Methods

### Analytical framework for equivalent elastic moduli of nano-heterostructures

**Figure 7:**
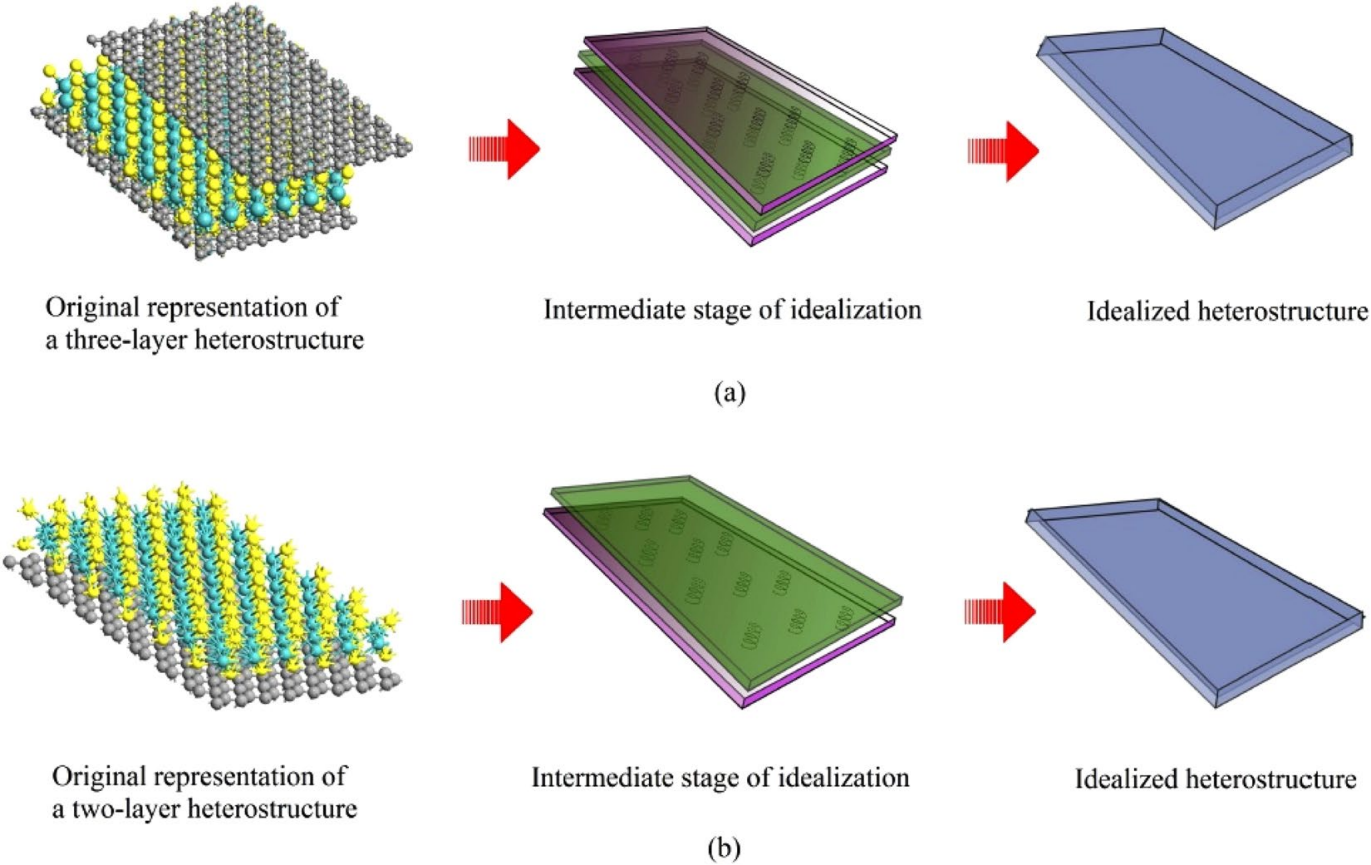
(**a**) Idealization scheme for the analysis of a three-layer nano-heterostructure. (**b**) Idealization scheme for the analysis of a two-layer nano-heterostructure.

A concise description of the basic philosophy behind the developed analytical framework is explained in this section (detail derivations are provided as supplementary material with this manuscript). A multi-stage bottom-up idealization scheme is adopted for deriving the closed-form expressions, as depicted in Fig. 7. In the first stage, the effective elastic moduli of each individual layer are determined based on a continuum based approach. This is equivalent to the effective elastic properties of a single-layer nanostructure. The multi-layer heterostructure can be idealized as a layered plate-like composite structural element with respective effective elastic properties and geometric dimensions (such as thickness) of each layer. To ensure the consistency in deformation of the adjacent layers, each of the layers are considered to have equal effective deformation in a particular direction. The equivalent elastic property of the entire heterostructure is determined based on force equilibrium and deformation compatibility conditions. The molecular mechanics parameters (*k*_*r*_ and *k*_*θ*_), bond length and bond angles for different materials, which are used to obtain numerical results based on Equations 3–6, are provided in the next paragraph.

The molecular mechanics parameters and geometric properties of the bonds are well-documented in scientific literature. In case of graphene, the molecular mechanics parameters *k*_*r*_ and *k*_*θ*_ can be obtained from literature using AMBER force filed^53^ as *k*_*r*_ = 938 kcal mol^−1^ nm^−2^ = 6.52 × 10^−7^ Nnm^−1^ and *k*_*θ*_ = 126 kcal mol^−1^ rad^−2^ = 8.76 × 10^−10^ Nnm rad^−2^. The out-of-plane angle for graphene is *α* = 0 and the bond angle is *θ* = 120° (i.e. *ψ* = 30°), while bond length and thickness of single-layer graphene can be obtained from literature as 0.142 nm and 0.34 nm respectively^7^. In case of hBN, the molecular mechanics parameters *k*_*r*_ and *k*_*θ*_ can be obtained from literature using DREIDING force model^54^ as *k*_*r*_ = 4.865 × 10^−7^ Nnm^−1^ and *k*_*θ*_ = 6.952 × 10^−10^ Nnm rad^−2^ ^55^. The out-of-plane angle for hBN is *α* = 0 and the bond angle is *θ* = 120° (i.e. *ψ* = 30°), while bond length and thickness of single-layer hBN can be obtained from literature as 0.145 nm and 0.098 nm respectively^8^. In case of stanene, the molecular mechanics parameters *k*_*r*_ and *k*_*θ*_ can be obtained from literature as *k*_*r*_ = 0.85 × 10^−7^ Nnm^−1^ and *k*_*θ*_ = 1.121 × 10^−9^ Nnm rad^−2^ ^56,57^. The out-of-plane angle for stanene is *α* = 17.5° and the bond angle is *θ* = 109° (i.e. *ψ* = 35.5°), while bond length and thickness of single layer stanene can be obtained from literature as 0.283 nm and 0.172 nm respectively^56–59^. In case of MoS_2_, the molecular mechanics parameters *k*_*r*_ and *k*_*θ*_ can be obtained from literature as *k*_*r*_ = 1.646 × 10^−7^ Nnm^−1^ and *k*_*θ*_ = 1.677 × 10^−9^ Nnm rad^−2^, while the out-of-plane angle, bond angle, bond length and thickness of single layer MoS_2_ are *α* = 48.15°, *θ* = 82.92° (i.e. *ψ* = 48.54°), 0.242 nm and 0.6033 nm respectively^15,60–62^.

### Molecular dynamics simulation for Poisson’s ratios of graphene–MoS_2_ heterostructures

We have followed a similar method as reported in literature^21,63,64^ for calculating the Poisson’s ratios of graphene–MoS_2_ bilayers and heterostructures through molecular dynamics simulation. The interatomic potential used for carbon-carbon, molybdenum-sulfur interactions are the second-generation Brenner interatomic potential^65,66^. We have stabilized the heterostructures following the same method as described in literature^21^. The MoS_2_ and graphene layers of the heterostructures are coupled by van der Waals interactions, as described by the Lennard-Jones potential. The adopted cut-off is 10.0*A*° for M/G/M and 5.0*A*° for G/M/G heterostructures. These cut-off values are determined by stabilizing and minimizing the M/G/M and G/M/G heterostructures^21^.

## Electronic supplementary material

Supplementary Information
