# Supplementary material for: Effective mechanical properties of multilayer nano-heterostructures
Source: Sci Rep. 2017 Nov 17;7:15818. doi: 10.1038/s41598-017-15664-3 (PMC5693924; doi:10.1038/s41598-017-15664-3)
Supplement: Supplementary file 1 — Supplementary Information [file 41598_2017_15664_MOESM1_ESM.pdf]

Supplementary material: Effective mechanical properties of multilayer nano-heterostructures

T. Mukhopadhyay[a], A. Mahata[b], S. Adhikari[c*], M. Asle Zaeem[b]

[a]*Department of Engineering Science, University of Oxford, Oxford, UK*
[b]*Department of Materials Science and Engineering, Missouri University of Science and Technology, Rolla, USA*
[c]*College of Engineering, Swansea University, Swansea, UK*

Generalized closed-form analytical formulae for the elastic moduli (in-plane Young's moduli and Poisson's ratios) of nano-heterostructures are developed based on the concept of linkage between the molecular mechanics parameters and structural mechanics parameters of the interatomic bonds. The molecular mechanics based approach for obtaining the equivalent elastic properties of atomic bonds is well-documented in scientific literature [1–3]. Therefore, the main contribution of this article lies in proposing computationally efficient and generalized analytical formulae for the elastic properties of nano-heterostructures.

1. Mechanical equivalence of atomic bonds

For atomic level behaviour of nano-scale materials, the total interatomic potential energy can be expressed as the sum of various individual energy terms related to bonding and non-bonding interactions [1]. Total strain energy (E) is expressed as the sum of energy contributions from bending of bonds (E_b), bond stretching (E_s), torsion of bonds (E_t) and energies associated with non-bonded terms (E_{nb}) such as the van der Waals attraction, the core repulsions and the coulombic energy (refer to figure 2 of the main manuscript).

$$E = E_s + E_b + E_t + E_{nb} \tag{1}$$

However, among all the energy components, effect of bending and stretching are predominant in case of small deformation [2, 3]. For the multiplanar hexagonal nano-structures (such as stanene and MoS_2), the strain energy caused by bending consists of two components, in-plane component (E_{bI}) and out-of-plane component (E_{bO}). The predominant deformation mechanisms for a multiplanar nanostructure are

*Corresponding author: S. Adhikari
Email: tanmoy.mukhopadhyay@eng.ox.ac.uk (T. Mukhopadhyay); s.adhikari@swansea.ac.uk (S. Adhikari)

depicted in figure 1 – 2. It can be noted that the out-of-plane component becomes zero for monoplanar nanostructures such as graphane and hBN. The total inter-atomic potential energy (E) can be expressed as

$$\begin{aligned} E &= E_s + E_{bI} + E_{bO} \\ &= \frac{1}{2}k_r(\Delta l)^2 + \left(\frac{1}{2}k_\theta(\Delta\theta)^2 + \frac{1}{2}k_\theta(\Delta\alpha)^2\right) \end{aligned} \tag{2}$$

where Δl, $\Delta\theta$ and $\Delta\alpha$ denote the change in bond length, in-plane and out-of-plane angle respectively, as shown in figure 1. The quantities k_r and k_θ are the force constants associated with bond stretching and bond bending respectively. The first term in Equation 2 corresponds to strain energy due to stretching (E_s), while the terms within bracket represent the strain energies due to in-plane (E_{bI}) and out-of-plane (E_{bO}) angle variations, respectively. The force constants of the atomic bonds (k_r and k_θ) can be expressed in the form of structural equivalence [4]. As per the standard theory of classical structural mechanics (refer to figure 2), strain energy of a uniform circular beam with cross-sectional area A, length l, Young's modulus E, and second moment of area I, under the application of a pure axial force N (refer to figure 2(b)) can be expressed as

$$U_a = \frac{1}{2}\int_0^L \frac{N^2}{EA}\,\mathrm{d}l = \frac{1}{2}\frac{N^2 l}{EA} = \frac{1}{2}\frac{EA}{l}(\Delta l)^2 \tag{3}$$

The strain energies due to pure bending moment M (refer to figure 2(c)) can be written as

$$U_b = \frac{1}{2}\int_0^L \frac{M^2}{EI}\,\mathrm{d}l = \frac{1}{2}\frac{EI}{l}(2\Delta\phi)^2 \tag{4}$$

Comparing Equation 3 with the expression for strain energy due to stretching (E_s) (refer Equation 2), it can be concluded that $K_r = \frac{EA}{l}$. For bending, it is reasonable to assume that $2\Delta\phi$ is equivalent to $\Delta\theta$ and $\Delta\alpha$ for in-plane and out-of-plane angle variations respectively (refer to figure 2(b)). Thus comparing Equation 4 with the expressions for the strain energies due to in-plane (E_{bI}) and out-of-plane (E_{bO}) angle variations, the following relation can be obtained: $k_\theta = \frac{EI}{l}$. On the basis of the established mechanical equivalence between molecular mechanics parameters (k_r and k_θ) and structural mechanics parameters (EA and EI), the effective elastic moduli of nano-heterostructures are obtained in the following subsection. Analytical formulae for elastic moduli of single-layer hexagonal nanostructures have been reported in literature [2, 5–8]; however, contribution of the present article is to develop closed-form analytical formulae for multi-layer nano-heterostructures. Equivalent elastic properties of the nano-heterostructures are derived based on a multi-stage bottom-up idealization scheme as depicted

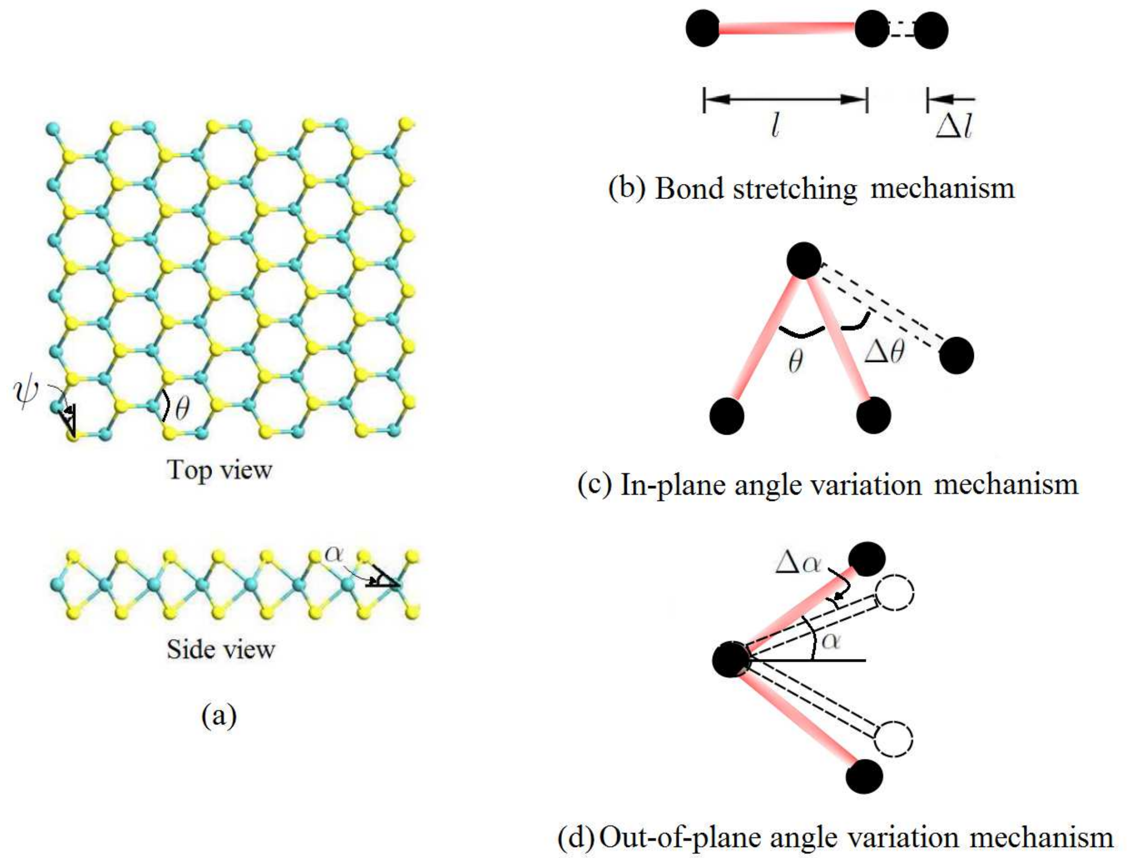

Figure 1: (a) Top view and side view of a multiplanar hexagonal nanostructure (b) Strain energy due to bond stretching (c) Strain energy due to in-plane angle variation (d) Strain energy due to out-of-plane angle variation

in figure 1M of the original manuscript. In the first stage, the effective elastic moduli of each individual layer are determined based on a mechanics based approach using the mechanical equivalence of bond properties. Thus the multi-layer heterostructure can be idealized as a layered plate-like structural element with respective effective elastic properties and geometric dimensions (such as thickness) of each layer. Each of the layers are considered to be bonded perfectly with adjacent layers. The equivalent elastic properties of the entire heterostructure is determined based on force equilibrium and deformation compatibility conditions at the final stage.

2. Young's modulus E_1 for nano-heterostructures

One generalized hexagonal unit cell is considered to derive the expression for Young's moduli of the entire hexagonal periodic nano-structure as shown in figure 2(a). Because of structural symmetry, horizontal deformation of the unit cell can be obtained by analysing the member AB only. The total

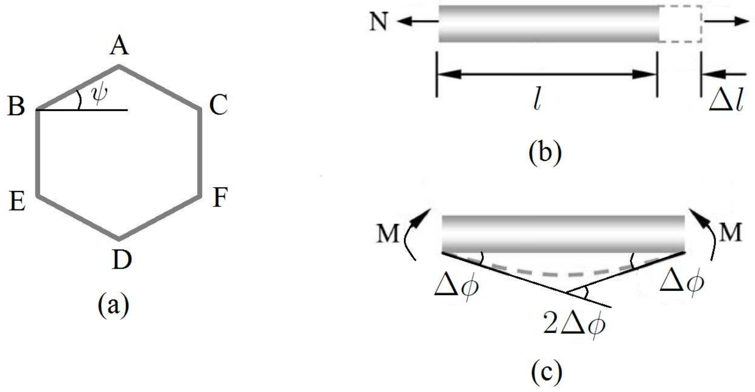

Figure 2: (a) Top view of a unit cell consisting of multiple idealized beam elements (refer to figure 1(a)) (b) A structural beam element subjected to pure tension (c) A structural beam element subjected to pure bending

horizontal deformation of the member AB (horizontal deflection of one end of the member with respect to the other end) under the application of stress σ_1 (refer to figure 3) has three components: axial deformation (δ_{aHi}), bending deformation due to in-plane loading (δ_{bHIi}) and bending deformation due to out-of-plane loading (δ_{bHOi}).

$$\begin{aligned} \delta_{H11i} &= \delta_{aHi} + \delta_{bHIi} + \delta_{bHOi} \\ &= \frac{H_i l_i \cos^2\psi_i \cos^2\alpha_i}{A_i E_i} + \frac{H_i l_i^3 \sin^2\psi_i}{12 E_i I_i} + \frac{H_i l_i^3 \cos^2\psi_i \sin^2\alpha_i}{12 E_i I_i} \end{aligned} \tag{5}$$

where $A_i = \dfrac{\pi d_i^2}{4}$, $I_i = \dfrac{\pi d_i^4}{64}$ and $H_i = \sigma_1 t_i l_i (1 + \sin\psi_i)\cos\alpha_i$. The quantities l_i and d_i represent the length and diameter of the member AB respectively. t_i is the thickness of single layer of such periodic structural form. The subscript i ($i = 1, 2, 3...n$; n is the total number of layers) is used here to denote the structural attributes, material properties and force components corresponding to i^{th} layer of the heterostructure. The three parts of Equation 5 are derived by considering the respective deformation components in direction-1. Figure 3(a) and figure 3(b) show the member AB using top-view and side-view respectively, wherein the horizontal load H acts at the node A in the 1-2 plane. Inclination angle of the member AB in the 1-2 plane and 1-3 plane are ψ_i and α_i respectively, as evident from the top view and side view shown in figure 3. Using the relationship between molecular mechanics parameters (k_r and k_θ) and structural mechanics parameters (EA and EI), form Equation 5, the expression for

strain in direction-1 (due to loading in direction-1) can be written as

$$\begin{aligned}\epsilon_{11i} &= \frac{\delta_{H11i}}{l_i \cos\psi_i \cos\alpha_i} \\ &= \frac{\sigma_1 t_i l_i (1+\sin\psi_i)}{l_i \cos\psi_i}\left(\frac{l_i^2}{12k_{\theta i}}\left(\sin^2\psi_i + \cos^2\psi_i \sin^2\alpha_i\right) + \frac{\cos^2\psi_i \cos^2\alpha_i}{k_{ri}}\right)\end{aligned} \tag{6}$$

On the basis of the basic definition of Young's modulus ($E = \dfrac{\sigma}{\epsilon}$), the closed-form expression for Young's modulus in direction-1 for a single-layer multiplanar nanostructure (stacked in the ith layer of

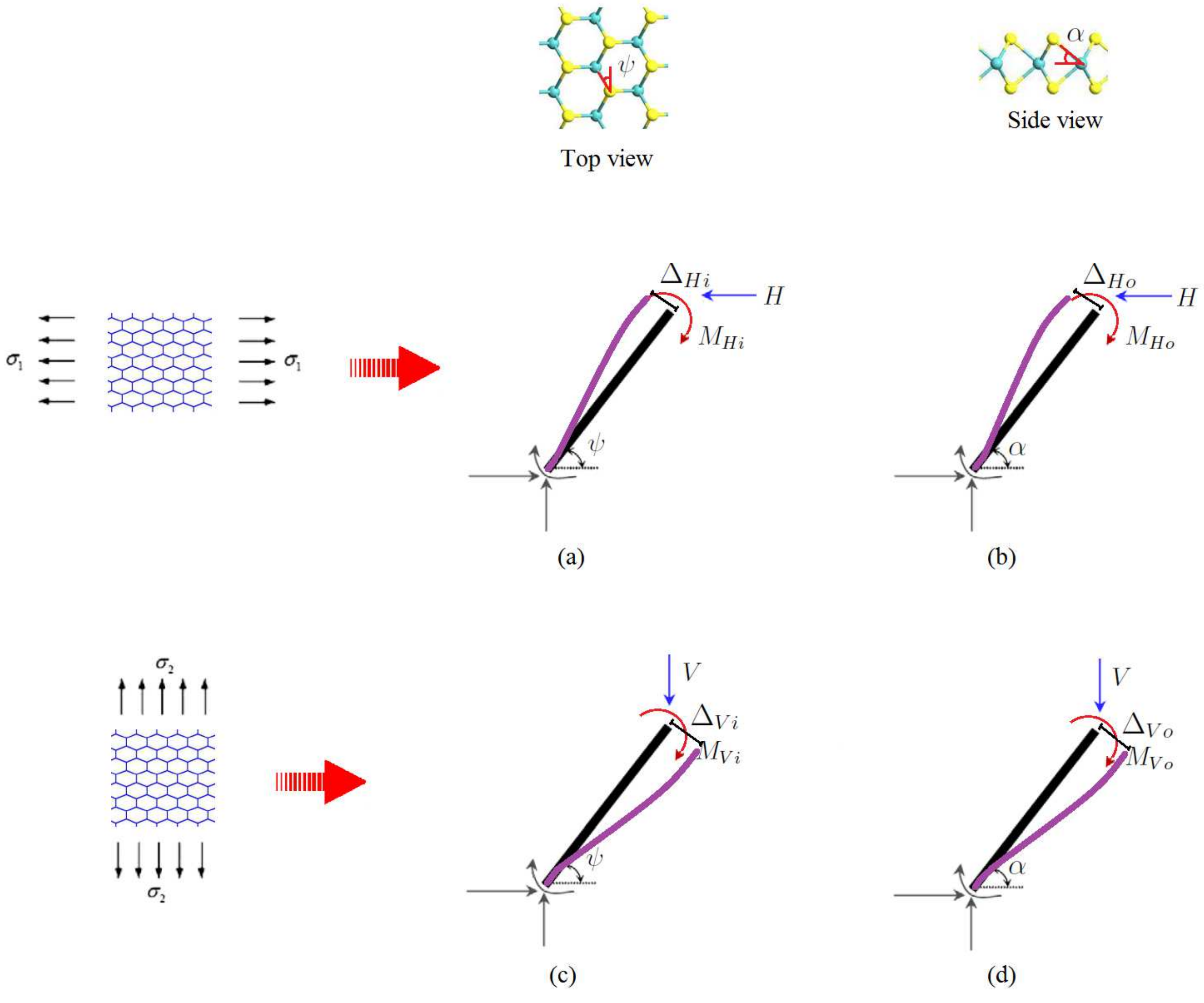

Figure 3: (a) Free body diagram of member AB for in-plane deformation under the application of horizontal force (b) Free body diagram of member AB for out-of-plane deformation under the application of horizontal force (c) Free body diagram of member AB for in-plane deformation under the application of vertical force (d) Free body diagram of member AB for out-of-plane deformation under the application of vertical force

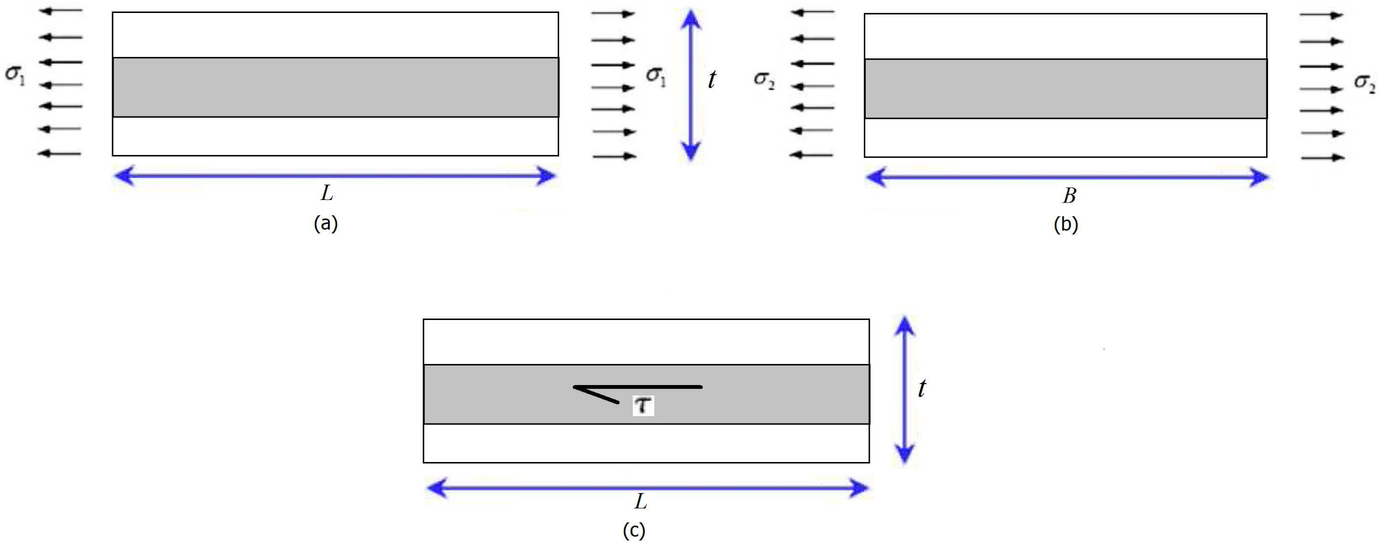

Figure 4: (a) Side view of the idealized heterostructure in $1-3$ plane along with applied stress in direction - 1 (b) Side view of the idealized heterostructure in $2-3$ plane along with applied stress in direction - 2 (c) Side view of the idealized heterostructure in $1-3$ plane along with applied shear stress

the heterostructure) can be obtained as

$$E_{1i} = \frac{\cos\psi_i}{t_i(1+\sin\psi_i)\left(\frac{l_i^2}{12k_{\theta i}}\left(\sin^2\psi_i + \cos^2\psi_i\sin^2\alpha_i\right) + \frac{\cos^2\psi_i\cos^2\alpha_i}{k_{ri}}\right)} \tag{7}$$

The subscript i in the above expression indicates the molecular mechanics and structural (/geometrical) properties corresponding to the ith layer.

Figure 4(a) shows the side view of an idealized three-layer heterostructure with the stress applied in direction - 1. It can be noted that we have show a three-layer heterostructure as an example; the derived formulae are applicable for heterostructures with any number of layers. From the condition of force equilibrium, the total force applied in direction - 1 should be equal to the summation of the force component shared by each of the constituting layers in the same direction. Thus considering a heterostructure with n number of layers

$$\sigma_1 tB = \sum_{i=1}^{n} \sigma_{1i} t_i B \tag{8}$$

where $t = \sum_{i=1}^{n} t_i$; t_i represents the thickness of ith layer ($i = 1, 2, 3...n$). From the definition of Young's modulus, the above expression can be written as

$$E_1 \epsilon_1 t = \sum_{i=1}^{n} E_{1i} \epsilon_{1i} t_i \tag{9}$$

where E_1 and ϵ_1 are the effective Young's modulus in direction - 1 and the strain in direction - 1 respectively for the entire heterostructure. E_{1i} and ϵ_{1i} represent the effective Young's modulus in direction - 1 and the strain in direction - 1 of ith layer respectively. As each of the layers are considered to have equal effective deformation in direction - 1, the deformation compatibility condition yields: $\epsilon_1 = \epsilon_{1i}, \in [1, n]$. Thus Equation 9 and 7 give the expression of Young's modulus in direction - 1 for the entire heterostructure as

$$\begin{aligned} E_1 &= \frac{1}{t}\sum_{i=1}^{n} E_{1i}t_i \\ &= \frac{1}{t}\sum_{i=1}^{n} \frac{\cos\psi_i}{(1+\sin\psi_i)\left(\frac{l_i^2}{12k_{\theta i}}\left(\sin^2\psi_i + \cos^2\psi_i \sin^2\alpha_i\right) + \frac{\cos^2\psi_i\cos^2\alpha_i}{k_{ri}}\right)} \end{aligned} \tag{10}$$

In the above expression $\psi_i = 90° - \frac{\theta_i}{2}$, where θ_i is the bond angle of the nanostructure stacked at i^{th} layer, as shown in figure 1(a). In case of a layer with monoplanar nanostructure, the out-of-plane angle α_i becomes zero.

3. Young's modulus E_2 for nano-heterostructures

Total vertical deformation of the unit cell (refer to figure 2(a)) under the application of σ_2 (refer to figure 3) is consisted of the deformation of member AB (δ_{V1i}) and member BE (δ_{V2i}) in direction-2. The deformed shapes and free body diagrams of the members under the application of vertical stresses are shown in figure 3(c – d). Deflection of joint A in direction-2 with respect to joint B has three components: axial deformation (δ_{aV1i}), bending deformation due to in-plane loading (δ_{bV1Ii}) and bending deformation due to out-of-plane loading (δ_{bV1Oi}).

$$\begin{aligned} \delta_{V1i} &= \delta_{aV1i} + \delta_{bV1Ii} + \delta_{bV1Oi} \\ &= \frac{V_i l_i \sin^2\psi_i \cos^2\alpha_i}{A_i E_i} + \frac{V_i l_i^3 \cos^2\psi_i}{12E_i I_i} + \frac{V_i l_i^3 \sin^2\psi_i \sin^2\alpha_i}{12E_i I_i} \end{aligned} \tag{11}$$

where $V_i = \sigma_2 t_i l_i \cos\psi_i \cos\alpha_i$. As the member BE is parallel to the 2-3 plane, deflection of joint B with respect to the joint E has two components: axial deformation (δ_{aV2i}) and bending deformation due to out-of-plane loading (δ_{bV2Oi}). It can be noted that the force acting on the member BE is $2V$ as there are similar unit cells adjacent to the one being analysed.

$$\begin{aligned} \delta_{V2i} &= \delta_{aV2i} + \delta_{bV2Oi} \\ &= \frac{2V_i l_i \cos^2\alpha_i}{A_i E_i} + \frac{2V_i l_i^3 \sin^2\alpha_i}{12E_i I_i} \end{aligned} \tag{12}$$

Replacing the expressions of V_i, A_i, I_i, A_iE_i and E_iI_i, the total deformation in direction-2 can be obtained from Equation 11 and 12 as

$$\begin{aligned}\delta_{V22i} &= \delta_{V1i} + \delta_{V2i} \\ &= \sigma_2 t_i l_i \cos\psi_i \cos\alpha_i \left(\frac{l_i^2}{12k_{\theta i}} \left(\cos^2\psi_i + \sin^2\psi_i \sin^2\alpha_i + 2\sin^2\alpha_i\right) + \frac{\cos^2\alpha_i}{k_{ri}} \left(\sin^2\psi_i + 2\right) \right)\end{aligned} \tag{13}$$

From Equation 13, the strain in direction-2 (due to loading in direction-2) can be expresses as

$$\begin{aligned}\epsilon_{22i} &= \frac{\delta_{V22i}}{(l_i + l_i \sin\psi_i)\cos\alpha_i} \\ &= \frac{\sigma_2 t_i \cos\psi_i}{1+\sin\psi_i} \left(\frac{l_i^2}{12k_{\theta i}} \left(\cos^2\psi_i + \sin^2\psi_i \sin^2\alpha_i + 2\sin^2\alpha_i\right) + \frac{\cos^2\alpha_i}{k_{ri}} \left(\sin^2\psi_i + 2\right) \right)\end{aligned} \tag{14}$$

On the basis of the basic definition of Young's modulus ($E = \dfrac{\sigma}{\epsilon}$), the closed-form expression for Young's modulus in direction-2 for a single-layer multiplanar nanostructure (stacked in the ith layer of the heterostructure) can be obtained as

$$E_{2i} = \frac{1+\sin\psi_i}{t_i \cos\psi_i \left(\frac{l_i^2}{12k_{\theta i}} \left(\cos^2\psi_i + \sin^2\psi_i \sin^2\alpha_i + 2\sin^2\alpha_i\right) + \frac{\cos^2\alpha_i}{k_{ri}} \left(\sin^2\psi_i + 2\right) \right)} \tag{15}$$

The subscript i in the above expression indicates the molecular mechanics and structural (/geometrical) properties corresponding to the ith layer.

Figure 4(b) shows the side view of an idealized three-layer heterostructure with the stress applied in direction - 2. From the condition of force equilibrium, the total force applied in direction - 2 should be equal to the summation of the force component shared by each of the constituting layers in the same direction. Thus considering a heterostructure with n number of layers

$$\sigma_2 t L = \sum_{i=1}^{n} \sigma_{2i} t_i L \tag{16}$$

where $t = \sum_{i=1}^{n} t_i$; t_i represents the thickness of ith layer ($i = 1, 2, 3...n$). From the definition of Young's modulus, the above expression can be written as

$$E_2 \epsilon_2 t = \sum_{i=1}^{n} E_{2i} \epsilon_{2i} t_i \tag{17}$$

where E_2 and ϵ_2 are the effective Young's modulus in direction - 2 and the strain in direction - 2 respectively for the entire heterostructure. E_{2i} and ϵ_{2i} represent the effective Young's modulus in direction - 2 and the strain in direction - 2 of ith layer respectively. As each of the layers are considered to have equal effective deformation in direction - 2, the deformation compatibility condition yields:

$\epsilon_2 = \epsilon_{2i}, \in [1, n]$. Thus Equation 17 and 15 give the expression of Young's modulus in direction - 2 for the entire heterostructure as

$$\begin{aligned} E_2 &= \frac{1}{t}\sum_{i=1}^{n} E_{2i} t_i \\ &= \frac{1}{t}\sum_{i=1}^{n} \frac{1+\sin\psi_i}{\cos\psi_i\left(\frac{l_i^2}{12k_{\theta i}}\left(\cos^2\psi_i + \sin^2\psi_i\sin^2\alpha_i + 2\sin^2\alpha_i\right) + \frac{\cos^2\alpha_i}{k_{ri}}\left(\sin^2\psi_i + 2\right)\right)} \end{aligned} \tag{18}$$

In the above expression $\psi_i = 90^\circ - \dfrac{\theta_i}{2}$, where θ_i is the bond angle of the nanostructure stacked at i^{th} layer, as shown in figure 1(a). In case of a layer with monoplanar nanostructure, the out-of-plane angle α_i becomes zero.

4. In-plane Poisson's ratio ν_{12} for nano-heterostructures

In-plane Poisson's ratio for the loading direction-1 (ν_{12i}) can be obtained as

$$\nu_{12i} = -\frac{\epsilon_{12i}}{\epsilon_{11i}} \tag{19}$$

where ϵ_{12i} and ϵ_{11i} are the strains in direction-2 and direction-1 respectively due to loading in direction-1. The expression for ϵ_{11i} is given in Equation 6. Derivation for the expression of ϵ_{12i} is provided next. The deformation in direction-2 due to loading in direction-1 can be obtained by considering one hexagonal unit cell as shown in figure 2(a). Because of structural symmetry, deformation in direction-2 of the unit cell due to loading in direction-1 can be obtained by analysing the member AB only. The total deformation in direction-2 of the member AB (deflection in direction-2 of one end of the member with respect to the other end) under the application of stress σ_1 has two components (refer to figure 3(a-b)): bending deformation due to in-plane loading (δ_{bVI1i}) and bending deformation due to out-of-plane loading (δ_{bVO1i}).

$$\begin{aligned} \delta_{H12i} &= \delta_{bVI1i} + \delta_{bVO1i} \\ &= -\frac{H_i l_i^3 \sin\psi_i\cos\psi_i}{12E_iI_i} + \frac{H_i l_i^3 \sin\psi_i\cos\psi_i\sin^2\alpha_i}{12E_iI_i} \\ &= -\frac{H_i l_i^3 \sin\psi_i\cos\psi_i\cos^2\alpha_i}{12E_iI_i} \end{aligned} \tag{20}$$

Using the relationship between molecular mechanics parameter k_θ and structural mechanics parameter EI, form Equation 20, the expression for strain in direction-2 (due to loading in direction-1) can be

written as

$$\begin{aligned}\epsilon_{12i} &= \frac{\delta_{H12i}}{(l_i + l_i \sin\psi_i)\cos\alpha_i} \\ &= -\frac{H_i l_i \sin\psi_i \cos\psi_i \cos\alpha_i}{12k_{\theta i}\,(1+\sin\psi_i)}\end{aligned} \tag{21}$$

On the basis of the basic definition of ν_{12i} as shown in Equation 19, the closed-form expression of in-plane Poisson's ratio for the loading direction-1 in case of a single-layer multiplanar nanostructure (stacked in i^{th} layer of the heterostructure) can be obtained as

$$\nu_{12i} = \frac{\sin\psi_i \cos^2\psi_i \cos^2\alpha_i l_i^2}{12k_{\theta i}\,(1+\sin\psi_i)\left(\dfrac{l_i^2}{12k_{\theta i}}\left(\sin^2\psi_i + \cos^2\psi_i \sin^2\alpha_i\right) + \dfrac{\cos^2\psi_i \cos^2\alpha_i}{k_{ri}}\right)} \tag{22}$$

The subscript i in the above expression indicates the molecular mechanics and structural (/geometrical) properties corresponding to the ith layer.

From Equation 9, using the basic definition of ν_{12} for the entire heterostructure and also the individual layers we get

$$E_1 \frac{\epsilon_2}{\nu_{12}} t = \sum_{i=1}^{n} E_{1i} \frac{\epsilon_{2i}}{\nu_{12i}} t_i \tag{23}$$

As each of the layers are considered to have equal effective deformation, the deformation compatibility condition yields: $\epsilon_2 = \epsilon_{2i}, \in [1, n]$. Thus, from Equation 23 and Equation 10, we have

$$\begin{aligned}\nu_{12} &= \frac{E_1 t}{\displaystyle\sum_{i=1}^{n} \frac{E_{1i} t_i}{\nu_{12i}}} \\ &= \frac{\displaystyle\sum_{i=1}^{n} E_{1i} t_i}{\displaystyle\sum_{i=1}^{n} \frac{E_{1i} t_i}{\nu_{12i}}}\end{aligned} \tag{24}$$

Based on Equation 22 and Equation 10, the above equation give the expression of in-plane Poisson's ratio of a nano-heterostructure for loading direction - 1 as

$$\nu_{12} = \frac{\displaystyle\sum_{i=1}^{n} \frac{\cos\psi_i}{(1+\sin\psi_i)\left(\dfrac{l_i^2}{12k_{\theta i}}\left(\sin^2\psi_i + \cos^2\psi_i \sin^2\alpha_i\right) + \dfrac{\cos^2\psi_i \cos^2\alpha_i}{k_{ri}}\right)}}{\displaystyle\sum_{i=1}^{n} \frac{12k_{\theta i}}{\sin\psi_i \cos\psi_i \cos^2\alpha_i l_i^2}} \tag{25}$$

In the above expression $\psi_i = 90° - \dfrac{\theta_i}{2}$, where θ_i is the bond angle of the nanostructure stacked at i^{th}

layer, as shown in figure 1(a). In case of a layer with monoplanar nanostructure, the out-of-plane angle α_i becomes zero.

5. In-plane Poisson's ratio ν_{21} for nano-heterostructures

In-plane Poisson's ratio for the loading direction-2 (ν_{21i}) can be obtained as

$$\nu_{21i} = -\frac{\epsilon_{21i}}{\epsilon_{22i}} \tag{26}$$

where ϵ_{21i} and ϵ_{22i} are the strains in direction-1 and direction-2 respectively due to loading in direction-2. The expression for ϵ_{22i} is given in Equation 14. Derivation for the expression of ϵ_{21i} is provided next. The deformation in direction-1 due to loading in direction-2 can be obtained by considering one hexagonal unit cell as shown in figure 2(a). Because of structural symmetry, deformation in direction-1 of the unit cell due to loading in direction-2 can be obtained by analysing the member AB only. The total deformation in direction-1 of the member AB (deflection in direction-1 of one end of the member with respect to the other end) under the application of stress σ_2 has two components (refer to figure 3(c - d)): bending deformation due to in-plane loading (δ_{bHI2i}) and bending deformation due to out-of-plane loading (δ_{bHO2i}).

$$\begin{aligned}\delta_{H21i} &= \delta_{bHI2i} + \delta_{bHO2i} \\ &= -\frac{V_i l_i^3 \sin\psi_i \cos\psi_i}{12E_iI_i} + \frac{V_i l_i^3 \sin\psi_i \cos\psi_i \sin^2\alpha_i}{12E_iI_i} \\ &= -\frac{V_i l_i^3 \sin\psi_i \cos\psi_i \cos^2\alpha_i}{12E_iI_i}\end{aligned} \tag{27}$$

Using the relationship between molecular mechanics parameter k_θ and structural mechanics parameter EI, form Equation 27, the expression for strain in direction-1 (due to loading in direction-2) can be written as

$$\begin{aligned}\epsilon_{21i} &= \frac{\delta_{H21i}}{l_i \cos\psi_i \cos\alpha_i} \\ &= -\frac{V_i l_i \sin\psi_i \cos\alpha_i}{12k_{\theta_i}}\end{aligned} \tag{28}$$

On the basis of the basic definition of ν_{21i} as shown in Equation 26, the closed-form expression of in-plane Poisson's ratio for the loading direction-2 in case of a single-layer multiplanar nanostructure (stacked in i^{th} layer of the heterostructure) can be obtained as

$$\nu_{21i} = \frac{\sin\psi_i (1+\sin\psi_i)\cos^2\alpha_i l_i^2}{12k_{\theta i}\left(\frac{l_i^2}{12k_{\theta i}}\left(\cos^2\psi_i + \sin^2\psi_i \sin^2\alpha_i + 2\sin^2\alpha_i\right) + \frac{\cos^2\alpha_i}{k_{ri}}\left(\sin^2\psi_i + 2\right)\right)} \tag{29}$$

The subscript i in the above expression indicates the molecular mechanics and structural (/geometrical) properties corresponding to the ith layer.

From Equation 17, using the basic definition of ν_{21} for the entire heterostructure and also the individual layers we get

$$E_2 \frac{\epsilon_1}{\nu_{21}} t = \sum_{i=1}^{n} E_{2i} \frac{\epsilon_{1i}}{\nu_{21i}} t_i \tag{30}$$

As each of the layers are considered to have equal effective deformation, the deformation compatibility condition yields: $\epsilon_1 = \epsilon_{1i}, \in [1, n]$. Thus, from Equation 30 and 18, we have

$$\begin{aligned} \nu_{21} &= \frac{E_2 t}{\sum_{i=1}^{n} \frac{E_{2i} t_i}{\nu_{21i}}} \\ &= \frac{\sum_{i=1}^{n} E_{2i} t_i}{\sum_{i=1}^{n} \frac{E_{2i} t_i}{\nu_{21i}}} \end{aligned} \tag{31}$$

Based on equation 29 and 18, the above equation give the expression of in-plane Poisson's ratio of a nano-heterostructure for loading direction - 2 as

$$\nu_{21} = \frac{\sum_{i=1}^{n} \frac{1 + \sin\psi_i}{\cos\psi_i \left(\frac{l_i^2}{12k_{\theta i}} \left(\cos^2\psi_i + \sin^2\psi_i \sin^2\alpha_i + 2\sin^2\alpha_i\right) + \frac{\cos^2\alpha_i}{k_{ri}} \left(\sin^2\psi_i + 2\right) \right)}}{\sum_{i=1}^{n} \frac{12k_{\theta i}}{\sin\psi_i \cos\psi_i \cos^2\alpha_i l_i^2}} \tag{32}$$

In the above expression $\psi_i = 90° - \frac{\theta_i}{2}$, where θ_i is the bond angle of the nanostructure stacked at i^{th} layer, as shown in figure 1(a). In case of a layer with monoplanar nanostructure, the out-of-plane angle α_i becomes zero.

Thus the in-plane Young's moduli and Poisson's ratios of multi-layer nano-heterostructures as well as single-layer hexagonal nanostructures can be predicted using the closed-form formulae derived in this article from molecular mechanics parameters (k_r and k_θ), bond length (l), bond angle (θ) and out-of-plane angle (α), which are well-documented in the molecular mechanics literature.

6. Remark on single-layer nanostructures

The closed-form analytical formulae derived for the elastic moduli of multi-layer nano-heterostructures reduce to the expressions for single-layer multiplanar nanostructures in case of $n = 1$.

$$E_1 = \frac{\cos\psi}{t(1+\sin\psi)\left(\frac{l^2}{12k_\theta}\left(\sin^2\psi+\cos^2\psi\sin^2\alpha\right)+\frac{\cos^2\psi\cos^2\alpha}{k_r}\right)} \tag{33}$$

$$E_2 = \frac{1+\sin\psi}{t\cos\psi\left(\frac{l^2}{12k_\theta}\left(\cos^2\psi+\sin^2\psi\sin^2\alpha+2\sin^2\alpha\right)+\frac{\cos^2\alpha}{k_r}\left(\sin^2\psi+2\right)\right)} \tag{34}$$

$$\nu_{12} = \frac{\sin\psi\cos^2\psi\cos^2\alpha l^2}{12k_\theta\left(1+\sin\psi\right)\left(\frac{l^2}{12k_\theta}\left(\sin^2\psi+\cos^2\psi\sin^2\alpha\right)+\frac{\cos^2\psi\cos^2\alpha}{k_r}\right)} \tag{35}$$

$$\nu_{21} = \frac{\sin\psi\left(1+\sin\psi\right)\cos^2\alpha l^2}{12k_\theta\left(\frac{l^2}{12k_\theta}\left(\cos^2\psi+\sin^2\psi\sin^2\alpha+2\sin^2\alpha\right)+\frac{\cos^2\alpha}{k_r}\left(\sin^2\psi+2\right)\right)} \tag{36}$$

The above expressions for single-layer nanostructures perfectly obey the Reciprocal theorem implying that only (any) three of the four elastic moduli E_1, E_2, ν_{12} and ν_{21} are independent.

$$E_1\nu_{21} = E_2\nu_{12} = \frac{\sin\psi\cos\psi\cos^2\alpha l^2\left(\frac{l^2}{12k_\theta}\left(\sin^2\psi+\cos^2\psi\sin^2\alpha\right)+\frac{\cos^2\psi\cos^2\alpha}{k_r}\right)^{-1}}{12k_\theta t\left(\frac{l^2}{12k_\theta}\left(\cos^2\psi+\sin^2\psi\sin^2\alpha+2\sin^2\alpha\right)+\frac{\cos^2\alpha}{k_r}\left(\sin^2\psi+2\right)\right)} \tag{37}$$

In case of monoplanar nanostructures (such as graphene and hBN), the out-of-plane angle α becomes zero and subsequently the elastic moduli for single-layer nanostructures simplifies to the following forms

$$E_1 = \frac{\cos\psi}{t(1+\sin\psi)\left(\frac{l^2}{12k_\theta}\sin^2\psi+\frac{\cos^2\psi}{k_r}\right)} \tag{38}$$

$$E_2 = \frac{1+\sin\psi}{t\cos\psi\left(\frac{l^2}{12k_\theta}\cos^2\psi+\frac{\left(\sin^2\psi+2\right)}{k_r}\right)} \tag{39}$$

$$\nu_{12} = \frac{\sin\psi\cos^2\psi l^2}{12k_\theta\left(1+\sin\psi\right)\left(\frac{l^2}{12k_\theta}\sin^2\psi+\frac{\cos^2\psi}{k_r}\right)} \tag{40}$$

$$\nu_{21} = \frac{\sin\psi\,(1+\sin\psi)\,l^2}{12k_\theta\left(\dfrac{l^2}{12k_\theta}\cos^2\psi + \dfrac{\left(\sin^2\psi+2\right)}{k_r}\right)} \tag{41}$$

It can be noticed that for regular single-layer monoplanar nanostructures ($\psi = 30°$) such as graphene, $E_1 = E_2$ and $\nu_{12} = \nu_{21}$. The expressions obtained for Young's moduli in case of $\psi = 30°$ exactly matches with the formulae provided by Shokrieh and Rafiee [2] for graphene.

7. Effect of inter-layer stiffness contribution

We have investigated the effect of Lennard-Jones potentials in heterostructures by comparing their relative contribution to stiffness with that of the constituting layers (such as graphene or MoS2). The elastic moduli of a heterostructure are directly dependent on the stiffness components of the individual constituting layers and inter-layer parameters. The Lennard-Jones potential is a mathematical model that approximates the interaction between a pair of neutral atoms or molecules. This potential can be expressed as [9]

$$V_{ij} = \epsilon\left(\left(\frac{r_m}{r_m+r}\right)^{12} - 2\left(\frac{r_m}{r_m+r}\right)^{6}\right) \tag{42}$$

where $r_m = 2^{\frac{1}{6}}\sigma$ and $\sigma = \left(\dfrac{B}{A}\right)^{\frac{1}{6}}$. The quantity ϵ is given by $\left(\dfrac{A^2}{4B}\right)$. A and B are the attractive and repulsive constants in the Lennard-Jones potential. r denotes the atomic displacement along the length between two atoms. The parameters of Lennard-Jones potentials for graphene-MoS_2 heterostructures are shown in Table 1. The equivalent axial force related to the Lennard-Jones potential acting between a pair of atoms (i,j) can be obtained as

$$F_{ij} = \frac{\partial V_{ij}}{\partial r} \tag{43}$$

It can be noted that Equation 43 gives a non-linear relation between force and deformation. Thus corresponding relationship between stiffness and deformation can be computed by dividing the force values by respective deformation. The deformation (Δ) and line of action of the force F_{ij} for a typical three-layer heterostructure is shown in the inset of figure 5.

Typical non-linear variation of stiffness with deformation for the possible combination of atoms in graphene-MoS_2 heterostructures is presented in figure 5. It is found that the effect of stiffness contributed by the consideration of Lennard-Jones potentials is negligible compared to stiffness of the

Table 1: Parameters of Lennard-Jones potentials for graphene-MoS_2 heterostructures

Interaction Type	ϵ (meV)	σ (Å)
C-C	2.96 [10]	3.40 [10]
C-M	3.325 [11]	2.82 [11]
C-S	7.355 [11]	3.22 [11]
S-S	1.19 [12]	3.59 [12]
M-S	2.49 [12]	3.16 [12]
M-M	2.43 [12]	2.72 [12]

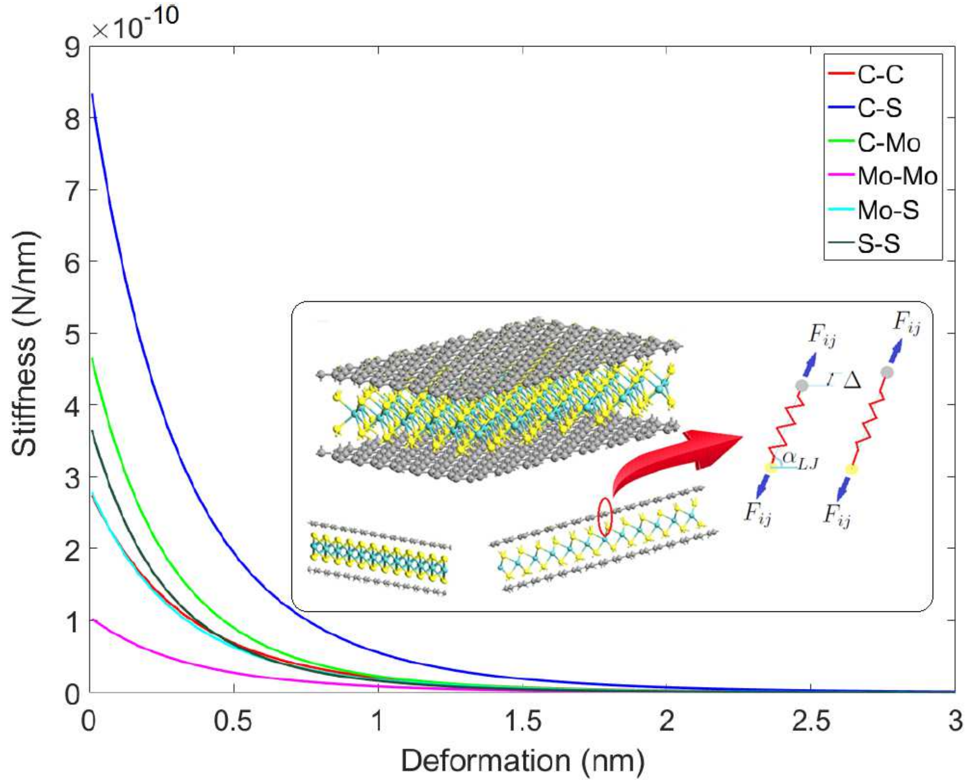

Figure 5: Variation of stiffness component due to Lennard-Jones potentials with deformation (pertaining to graphene-MoS_2 heterostructure). The deformation (Δ) and line of action of the force F_{ij} for a typical three-layer heterostructure is shown in the inset.

constituting layers of a heterostructure. In case of the graphene-MoS_2 heterostructure, the non-linear stiffness contribution due to Lennard-Jones potentials are found to be in the order of $\sim 10^{-10}$, while the stiffness of a single layer of graphene and MoS_2 (Young's modulus multiplied by respective single layer thickness) are in the order of $\sim 10^{-7}$ (refer to Table 1 of the main paper). Thus the incorporation of the

Table 2: Results for E_1 incorporating the effect of Lennard-Jones potentials (Here E_{1m} and E_{1c} represent the results obtained using equation 10 and equation 47 respectively)

Configuration	E_{1m}	E_{1c}				Error (%)			
		$p = 1$	$p = 0.9$	$p = 0.8$	$p = 0.7$	$p = 1$	$p = 0.9$	$p = 0.8$	$p = 0.7$
G/G	1.0419	1.0431	1.0430	1.0428	1.0427	0.1175	0.1058	0.0940	0.0823
M/M	0.1778	0.1785	0.1784	0.1784	0.1783	0.3870	0.3484	0.3098	0.2712
G/M	0.4893	0.4901	0.4901	0.4900	0.4899	0.1803	0.1623	0.1443	0.1263
M/G	0.4893	0.4901	0.4901	0.4900	0.4899	0.1803	0.1623	0.1443	0.1263
G/M/G	0.6357	0.6370	0.6368	0.6367	0.6366	0.2039	0.1836	0.1632	0.1428
M/G/M	0.3678	0.3689	0.3687	0.3686	0.3685	0.2922	0.2631	0.2339	0.2047

Table 3: Results for E_2 incorporating the effect of Lennard-Jones potentials (Here E_{2m} and E_{2c} represent the results obtained using equation 18 and equation 48 respectively)

Configuration	E_{2m}	E_{2c}				Error (%)			
		$p = 1$	$p = 0.9$	$p = 0.8$	$p = 0.7$	$p = 1$	$p = 0.9$	$p = 0.8$	$p = 0.7$
G/G	1.0419	1.0431	1.0430	1.0428	1.0427	0.1175	0.1058	0.0940	0.0823
M/M	0.3549	0.3556	0.3555	0.3555	0.3554	0.1942	0.1749	0.1555	0.1361
G/M	0.6025	0.6034	0.6033	0.6032	0.6031	0.1464	0.1318	0.1172	0.1025
M/G	0.6025	0.6034	0.6033	0.6032	0.6031	0.1464	0.1318	0.1172	0.1025
G/M/G	0.7189	0.7202	0.7201	0.7200	0.7198	0.1804	0.1624	0.1443	0.1263
M/G/M	0.5059	0.5070	0.5069	0.5068	0.5067	0.2126	0.1914	0.1701	0.1489

stiffness contribution due to Lennard-Jones potentials in the analytical formulation of heterostructures will marginally affect the final results. This is evident from the comparative results presented for graphene-MoS_2 heterostructure in Table 1 of the main paper, wherein a good agreement can be noticed between the analytical approach and molecular dynamics simulation. For this reason, we have not included the effect of Lennard-Jones potentials in the analytical formulation presented in the main paper. However, The contribution of stiffness due to Lennard-Jones potentials can be readily included in the proposed expression for elastic moduli. Representative effects of such additional stiffness contribution and corresponding error values for the two Young's moduli are presented in the following paragraph.

The modified expression of Young's modulus E_1 due to the consideration of additional stiffness contribution arising from the inter-layer interaction (r_s) can be derived by including the component

of force shared by the inter-layer regions. In a heterostructure with n layers, there would be $(n-1)$ numbers of inter-layer regions. Considering the equivalent in-plane stiffness of each inter-layer region as r_s, the modified condition of force equilibrium can be expressed as

$$\sigma_1 t B = \sum_{i=1}^{n} \sigma_{1i} t_i B + (n-1)\sigma_s t_s B \tag{44}$$

Here $\sigma_s t_s B$ is the in-plane force component shared by each of the inter-layer regions, wherein the equivalent stress and thickness of the inter-layer regions are denoted by σ_s and t_s, respectively. From the definition of Young's modulus, the above expression can be written as

$$E_1 \epsilon_1 t = \sum_{i=1}^{n} E_{1i} \epsilon_{1i} t_i + (n-1) E_s \epsilon_s t_s \tag{45}$$

where E_s and ϵ_s are the effective Young's modulus and the strain in direction - 1 respectively for the inter-layer regions. The deformation compatibility condition yields: $\epsilon_1 = \epsilon_{1i} = \epsilon_s, \in [1, n]$. Thus the above equation can be expressed as

$$E_1 t = \sum_{i=1}^{n} E_{1i} t_i + (n-1) r_s \tag{46}$$

where $r_s (= E_s \epsilon_s)$ is the in-plane stiffness contribution of the inter-layer regions arising due to Lennard-Jones potentials. The inter-layer regions of a heterostructure, as shown in figure 7 of the main manuscript using spring elements, can be idealized as an equivalent plate-like structure having an in-plane stiffness r_s. The springs in the figure 7 are typical representation of the stiffness component arising due to the effect described in Equation 43. Here it can be noted that the line of action of the stiffness components due to Lennard-Jones potentials is same as the line joining between two atoms of the adjacent layers. The contribution of stiffness along the in-plane direction would be a cosine component as shown in the inset of figure 5. Thus the effective in-plane stiffness of the inter-layer regions would always be lesser than the values reported in figure 5. The reduction in stiffness is accounted by introducing a fraction (p) for obtaining numerical results later in this section.

The modified expression of Young's modulus in direction - 1 for the entire heterostructure can be

derived from equation 7 and 46

$$\begin{aligned} E_{1c} &= \frac{1}{t}\sum_{i=1}^{n} E_{1i}t_i + \frac{(n-1)r_s}{t} \\ &= \frac{1}{t}\left(\sum_{i=1}^{n} \frac{\cos\psi_i}{(1+\sin\psi_i)\left(\frac{l_i^2}{12k_{\theta i}}\left(\sin^2\psi_i + \cos^2\psi_i \sin^2\alpha_i\right) + \frac{\cos^2\psi_i\cos^2\alpha_i}{k_{ri}}\right)} + (n-1)r_s\right) \end{aligned} \tag{47}$$

Similarly, the modified expression for Young's modulus E_2 incorporating the interlayer stiffness contribution can be derived as

$$\begin{aligned} E_{2c} &= \frac{1}{t}\sum_{i=1}^{n} E_{2i}t_i + \frac{(n-1)r_s}{t} \\ &= \frac{1}{t}\left(\sum_{i=1}^{n} \frac{1+\sin\psi_i}{\cos\psi_i\left(\frac{l_i^2}{12k_{\theta i}}\left(\cos^2\psi_i + \sin^2\psi_i \sin^2\alpha_i + 2\sin^2\alpha_i\right) + \frac{\cos^2\alpha_i}{k_{ri}}\left(\sin^2\psi_i + 2\right)\right)} + (n-1)r_s\right) \end{aligned} \tag{48}$$

where n is the number of layers and r_s is the stiffness contribution arising due to Lennard-Jones potentials.

From figure 5 it can be discerned that the value of r_s for a graphne-MoS_2 heterostructure should be 8.335×10^{-10} N/nm, or less, as discussed in the preceding paragraph (depending on the stacking sequence of the heterostructure and deformation value). Thus we present numerical results for variation of the Young's moduli of graphne-MoS_2 heterostructures as a fraction (p) of the maximum stiffness (i.e. $r_s = 8.335 \times 10^{-10}p$). It can be noted that the maximum error in the values of Young's moduli, as calculated without the effect of Lennard-Jones potentials (Equation 10 and 18), will occur for $p = 1$. The expressions accounting the effect of Lennard-Jones potentials (Equation 47 and 48) reduce to the expression's of Young's moduli provided in the main paper (Equation 10 and 18) for $p = 0$. Table 2 and 3 show the effect of considering inter-layer stiffness r_s on the Young's moduli, wherein it is evident that even the maximum possible value of error is quite negligible. Thus the formulae proposed for heterostructures without considering the effect of inter-layer stiffness can be used with sufficient level of accuracy.

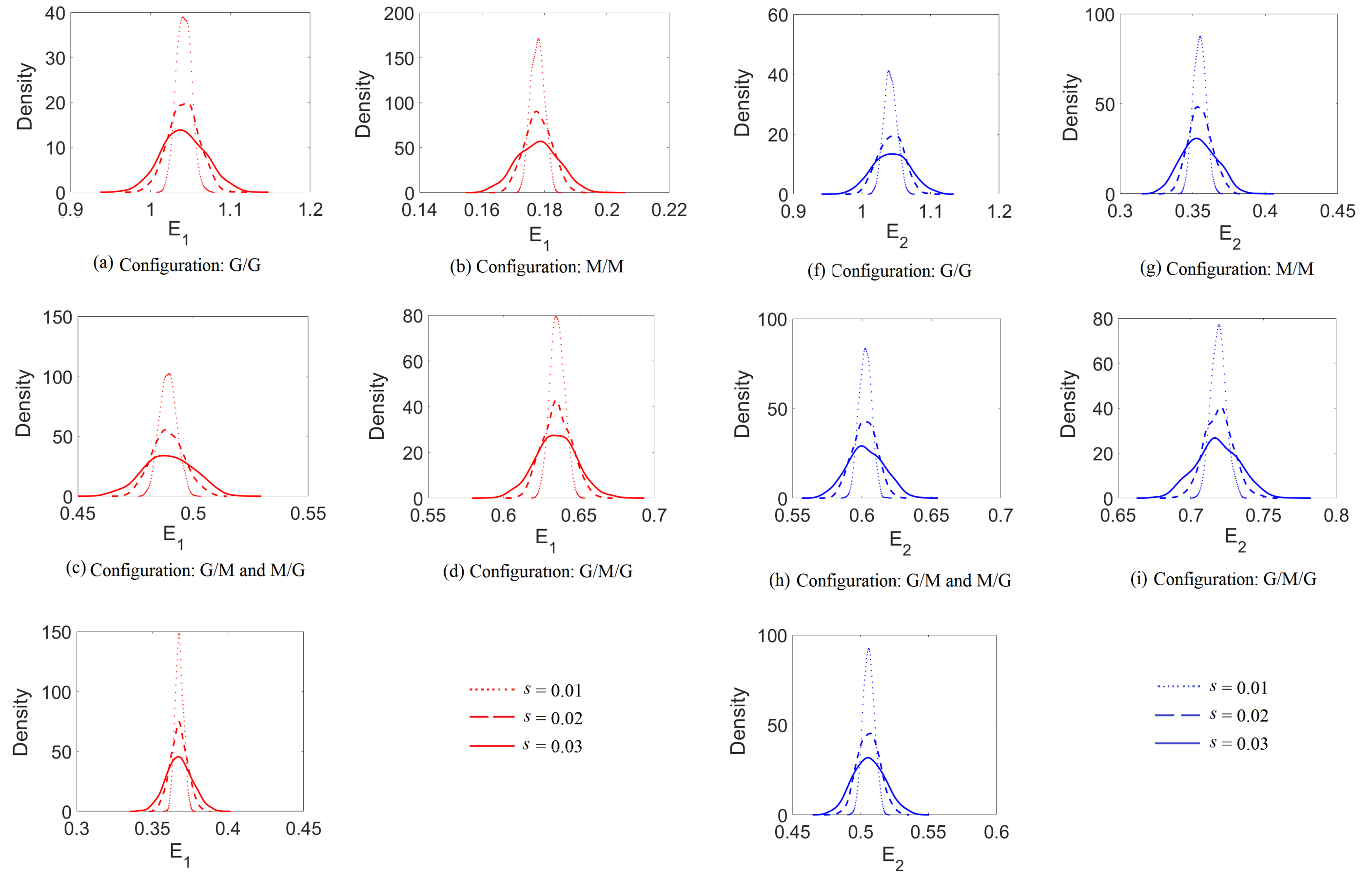

(a) Configuration: G/G
(b) Configuration: M/M
(c) Configuration: G/M and M/G
(d) Configuration: G/M/G
(e) Configuration: M/G/M
(f) Configuration: G/G
(g) Configuration: M/M
(h) Configuration: G/M and M/G
(i) Configuration: G/M/G
(j) Configuration: M/G/M

Figure 6: Effect of uncertainty on the Young's moduli (E_1 and E_2) of graphene-MoS_2 heterostructures

8. Effect of uncertainty on the elastic moduli

The proposed analytical closed-form formulae for the elastic moduli can be a quite efficient tool to account for uncertainty in the input parameters (following a Monte Carlo simulation based approach [13, 14]). The inevitable random variations in the input parameters can be considered analogous to the effect of epistemic uncertainty [15], which is quite relevant to the nanoscale analyses due to lack of precise knowledge regarding the nano-structures and related molecular mechanics parameters in many cases. Moreover, aleatoric uncertainty [15] may also be present in nanostructures due different forms of stochasticity in structural geometry. The effect of source uncertainty can be accounted in the elastic moduli as

$$E(s) = \Theta \left\{ \psi(s), \alpha(s), l(s), t(s), k_\theta(s), k_r(s) \right\} \tag{49}$$

Here E represents the elastic moduli such as Young's moduli and Poisson's ratios. Θ is a symbolic

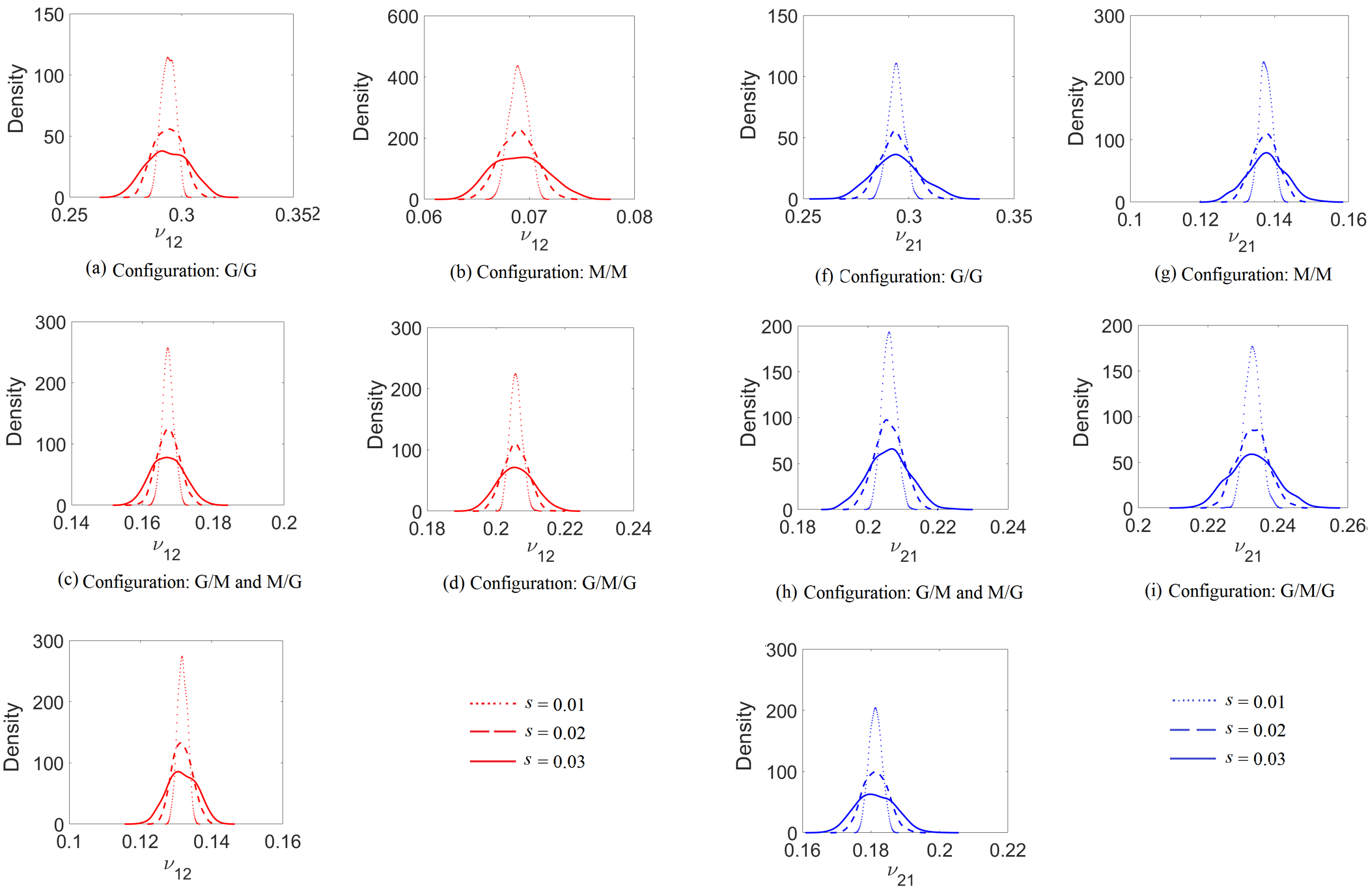

(a) Configuration: G/G (b) Configuration: M/M (f) Configuration: G/G (g) Configuration: M/M

(c) Configuration: G/M and M/G (d) Configuration: G/M/G (h) Configuration: G/M and M/G (i) Configuration: G/M/G

(e) Configuration: M/G/M (j) Configuration: M/G/M

Figure 7: Effect of uncertainty on the Poisson's ratios (ν_{12} and ν_{21}) of graphene-MoS_2 heterostructures

operator representing the Monte Carlo simulation considering the combined effect of all the stochastic input parameters, while s denotes the degree of stochasticity.

We have performed a Monte Carlo simulation based uncertainty analysis using the efficient analytical formulae to quantify the probabilistic characteristics of the elastic moduli of graphene-MoS_2 heterostructures (considering 10,000 simulations in each case). The probabilistic descriptions of the elastic moduli for three different levels of stochasticity (s) are presented in figure 6 and 7. It can be noticed that the response bounds increase with the increasing degree of stochasticity for all the heterostructures. Such probabilistic characterization can provide a comprehensive idea about the effect of source uncertainty on the elastic moduli of heterostructures.

References

[1] Chang, T. and Gao, H. Size-dependent elastic properties of a single-walled carbon nanotube via a molecular mechanics model. *Journal of the Mechanics and Physics of Solids*, 51(6):1059–1074, 2003.

[2] Shokrieh, M. M. and Rafiee, R. Prediction of young's modulus of graphene sheets and carbon nanotubes using nanoscale continuum mechanics approach. *Materials & Design*, 31:790–795, 2010.

[3] Gelin, B. R. *Molecular Modeling of Polymer Structures and Properties*. Hanser Gardner Publications, 1994.

[4] Li, C. and Chou, T. W. A structural mechanics approach for the analysis of carbon nanotubes. *International Journal of Solids and Structures*, 40(10):2487 – 2499, 2003.

[5] Scarpa, F., Adhikari, S., and Phani, A. S. Effective elastic mechanical properties of single layer graphene sheets. *Nanotechnology*, 20(6):065709, 2009.

[6] Mukhopadhyay, T., Mahata, A., Adhikari, S., and Zaeem, M. A. Effective elastic properties of two dimensional multiplanar hexagonal nanostructures. *2D Materials*, 4(2):025006, 2017.

[7] Boldrin, L., Scarpa, F., Chowdhury, R., and Adhikari, S. Effective mechanical properties of hexagonal boron nitride nanosheets. *Nanotechnology*, 22(50):505702, 2011.

[8] Le, M.-Q. Prediction of young's modulus of hexagonal monolayer sheets based on molecular mechanics. *International Journal of Mechanics and Materials in Design*, 11(1):15–24, 2015.

[9] Girifalco, L. A., Hodak, M., and Lee, R. S. Carbon nanotubes, buckyballs, ropes, and a universal graphitic potential. *Phys. Rev. B*, 62:13104–13110, 2000.

[10] Zhang, Y., Wang, C., Cheng, Y., and Xiang, Y. Mechanical properties of bilayer graphene sheets coupled by sp3 bonding. *Carbon*, 49(13):4511 – 4517, 2011.

[11] Liu, B., Meng, F., Reddy, C. D., Baimova, J. A., Srikanth, N., Dmitriev, S. V., and Zhou, K. Thermal transport in a graphene-mos2 bilayer heterostructure: a molecular dynamics study. *RSC Adv.*, 5:29193–29200, 2015.

[12] Ding, Z., Jiang, J.-W., Pei, Q.-X., and Zhang, Y.-W. In-plane and cross-plane thermal conductivities of molybdenum disulfide. *Nanotechnology*, 26(6):065703, 2015.

[13] Dey, S., Mukhopadhyay, T., and Adhikari, S. Metamodel based high-fidelity stochastic analysis of composite laminates: A concise review with critical comparative assessment. *Composite Structures*, 171:227 – 250, 2017.

[14] Dey, S., Mukhopadhyay, T., Sahu, S., and Adhikari, S. Effect of cutout on stochastic natural frequency of composite curved panels. *Composites Part B: Engineering*, 105:188 – 202, 2016.

[15] Dey, S., Mukhopadhyay, T., and Adhikari, S. Stochastic free vibration analysis of angle-ply composite plates – a rs-hdmr approach. *Composite Structures*, 122:526 – 536, 2015.
